# Reliability of pathophysiological markers reflective of exercise-induced gastrointestinal syndrome (EIGS) in response to 2-h high-intensity interval exercise: A comprehensive methodological efficacy exploration

**DOI:** 10.3389/fphys.2023.1063335

**Published:** 2023-02-21

**Authors:** Pascale Young, Isabella Russo, Paul Gill, Jane Muir, Rebekah Henry, Zoe Davidson, Ricardo J. S. Costa

**Affiliations:** ^1^ Department of Nutrition Dietetics and Food, Monash University, Notting Hill, VIC, Australia; ^2^ Department of Gastroenterology, Monash University, Melbourne, VIC, Australia; ^3^ Department of Civil Engineering, Monash University, Clayton, VIC, Australia

**Keywords:** running, I-FABP, endotoxin, bacteria, inflammation, gastrointestinal symptoms, short chain fatty acids

## Abstract

The study aimed to determine the test-retest reliability of exercise-induced gastrointestinal syndrome (EIGS) biomarkers, and assess the association of pre-exercise short chain fatty acid (SCFA) concentration with these biomarkers in response to prolonged strenuous exercise. Thirty-four participants completed 2 h of high-intensity interval training (HIIT) on two separate occasions with at least 5-days washout. Blood samples were collected pre- and post-exercise, and analysed for biomarkers associated with EIGS [i.e., cortisol, intestinal fatty-acid binding protein (I-FABP), sCD14, lipopolysaccharide binding protein (LBP), leukocyte counts, *in-vitro* neutrophil function, and systemic inflammatory cytokine profile]. Fecal samples were collected pre-exercise on both occasions. In plasma and fecal samples, bacterial DNA concentration was determined by fluorometer quantification, microbial taxonomy by 16S rRNA amplicon sequencing, and SCFA concentration by gas-chromatography. In response to exercise, 2 h of HIIT modestly perturbed biomarkers indicative of EIGS, including inducing bacteremia (i.e., quantity and diversity). Reliability analysis using comparative tests, Cohen’s *d*, two-tailed correlation, and intraclass correlation coefficient (ICC) of resting biomarkers presented good-to-excellent for IL-1ra (*r* = 0.710, ICC = 0.92), IL-10 (*r* = 0.665, ICC = 0.73), cortisol (*r* = 0.870, ICC = 0.87), and LBP (*r* = 0.813, ICC = 0.76); moderate for total (*r* = 0.839, ICC = 0.44) and per cell (*r* = 0.749, ICC = 0.54) bacterially-stimulated elastase release, IL-1β (*r* = 0.625, ICC = 0.64), TNF-α (*r* = 0.523, ICC = 0.56), I-FABP (*r* = 0.411, ICC = 0.21), and sCD14 (*r* = 0.409, ICC = 0.38), plus fecal bacterial α-diversity; and poor for leukocyte (*r* = 0.327, ICC = 0.33) and neutrophil (*r* = 0.352, ICC = 0.32) counts. In addition, a medium negative correlation was observed between plasma butyrate and I-FABP (*r* = −0.390). The current data suggest a suite of biomarkers should be used to determine the incidence and severity of EIGS. Moreover, determination of plasma and/or fecal SCFA may provide some insight into the mechanistic aspects of EIGS instigation and magnitude in response to exercise.

## Introduction

It is well established that exposure to prolonged strenuous exercise creates a reversible disturbance to the gastrointestinal tract through two predominant pathways. Firstly, the circulatory-gastrointestinal pathway, which describes redistribution of blood flow from the gastrointestinal tract to working muscle and peripheral circulation, to aid locomotion work and thermoregulation, respectively. Secondly, the neuroendocrine-gastrointestinal pathway, which describes the resulting increase in sympathetic drive, and therefore, a reduction in gastrointestinal function (Costa et al., 2017b; Costa R. J. S. et al., 2020). The combination of these gastrointestinal disturbances in response to exercise stress have been termed as “*exercise-induced gastrointestinal syndrome*” (EIGS), and are often linked to exercise-associated gastrointestinal symptoms (Ex-GIS) [e.g., belching, bloating, upper and lower abdominal bloating or pain, urge to regurgitate or defecate, regurgitation, abnormal defecation (excessive watery stools), and/or nausea]; which may manifest into performance impairment and/or health issues warranting medical attention and/or management (Costa et al., 2017a; Gaskell et al., 2019; Gaskell et al., 2021a; Gaskell et al., 2021b; Walter et al., 2021). Various extrinsic and intrinsic factors have been identified to exacerbate EIGS and Ex-GIS, as previously described and updated (Gaskell et al., 2021a; Costa et al., 2022). However, in the majority of studies such observations are predominantly limited to a narrow number of gastrointestinal integrity and/or functional variables, with none to modest magnitude in perturbations due to the limited exercise stress load (Costa et al., 2022). More recently, the impact of a longer (2 h) duration high-intensity interval exercise protocol, prompted increases in plasma variables indicative of intestinal epithelial integrity perturbations [Δ pre-to post-exercise intestinal fatty acid binding protein (I-FABP) +737 pg/ml, and sCD14 + 110 ng/ml], Ex-GIS (62% incidence and 8 arb. unit severity), and malabsorption of post-exercise recovery beverages (Russo et al., 2021a; Russo et al., 2021b; Russo et al., 2021c). In comparison steady state exercise for the same duration appears to elicit a more modest response in these biomarkers [e.g., 2 h continuous running at 60% *V̇*O_2_ (I-FABP +447 pg/ml, gram-negative bacterial endotoxin +4 pg/ml, Ex-GIS 70% incidence and 6 arb. unit severity), and 70% *V̇*O_2max_ (I-FABP +371 pg/ml, sCD14 + 53 ng/ml, Ex-GIS 64% incidence and 16 arb. unit severity)] when performed in temperate ambient conditions (Snipe and Costa, 2018b; Costa et al., 2019).

It is however important to highlight that a large individual variation in biomarkers consistent with gastrointestinal integrity perturbations was observed. Therefore, to date, the reliability of these EIGS variables at rest and in response to exercise protocols established to perturb gastrointestinal integrity and/or cause functional disturbance (i.e., 2 h high intensity interval exercise), plus induce Ex-GIS, is still relatively unknown.

A potent secondary outcome of EIGS, caused by epithelial injury and/or hyperpermeability of the circulatory-gastrointestinal pathway, is the potential for luminal originated microbial pathogenic agents to translocate into systemic circulation (Gill et al., 2015a; Gill et al., 2015b; Gill et al., 2016a), which may lead to systemic inflammatory responses, with or without clinical implications (Peake et al., 2015). Although there is substantial research exploring the impact of exercise stress on direct or indirect markers of bacterial endotoxin translocation [e.g., plasma lipopolysaccharides (LPS), LBP, sCD14, and/or EndoCAb concentration], research into exercise-associated whole bacterial luminal to systemic translocation (i.e., bacteremia) is scarce (Costa et al., 2022). Some previous studies have attempted to detect whole bacteria presence in circulation (e.g., total 16S bacteria: *Bacteroides* ratio) in response to prolonged low intensity exercise (i.e., 80 min fixed-intensity treadmill walking- 6 km/h and 7% gradient) (Ogden et al., 2020a; Ogden et al., 2020b), and higher intensity of shorter duration exercise (i.e., 60 min running at 70% *V̇*O_2peak_) (March et al., 2019; Ogden et al., 2022), with findings modest and inconsistent. Variability of bacterial translocation in response to these exercise bouts may be explained by the application of insufficient exertional stress among studies, a lack of control of confounding factors within experimental procedures, and/or the biomarker selected to represent whole bacterial translocation into systemic circulation (i.e., *Bacteriodes*/total bacterial DNA) (Costa et al., 2022).

No study to date has determined whether whole bacteria luminal translocation occurs in response to a more substantial and relevant exercise stress model reflective of real-life practices in athletes who frequently report gastrointestinal issues (i.e., endurance and team sports) (Gaskell et al., 2021a; Gaskell et al., 2021b). Furthermore, no study has reported the full plasma bacterial composition profile at rest and in response to exercise stress, as previously reported in both the clinical and exercise research arena (Castillo et al., 2019; Villarroel et al., 2022).

Research investigating short-chain fatty acids (SCFA) (i.e., butyrate, acetate, and propionate) and the potentially protective role these may play in gastrointestinal epithelial integrity, systemic responses, and impact on exercise performance, is attracting interest and gaining momentum (Clauss et al., 2021; Imdad et al., 2022). It has been demonstrated, albeit *in-vitro,* that bathing epithelial cell lines in concentrated SCFA solutions, particularly with butyrate, reduces permeability of epithelial cells (Mariadason et al., 1997). Thus, SCFA may have potential to attenuate exercise-associated epithelial perturbations, and subsequent systemic endotoxemia and/or bacteremia, which may flow onto reducing microbial translocation associated Ex-GIS incidence and severity (Gill et al., 2015a; Gill et al., 2015b; Gaskell et al., 2021b). Such protection may be attributed to enhanced epithelial cell (i.e., phospholipid bilayer) stability and/or tight-junction stability and regulation (Sekirov et al., 2010; Gilbert et al., 2018; Nabizadeh et al., 2022). There is evidence in human-exercise models to hypothesise that the presence of SCFA along the intestinal lumen, as a result of commensal microbial composition and function, may attenuate the effects of EIGS (Bennett et al., 2020). However, to date, no study has investigated whether an association exists between pre-exercise concentrations of luminal and systemic SCFAs and post-exercise markers of EIGS.

With this in mind, the current study primarily aimed to comprehensively determine the test-retest reliability of selected biomarkers linked to EIGS at rest prior to exercise and in response to prolonged strenuous exercise. In addition, a secondary aim was to assess the association between luminal and systemic SCFA concentration with these variables.

## Methods

### Participants

Thirty-four (n = 26 males, n = 8 females) recreationally competitive individuals exposed to endurance type training [mean (SD): Age 30 (8.0) years, nude body mass (NBM) 70.7 (10.3) kg, height 175 (9.0) cm, % body fat 15.9 (6.5) %, *V̇*O_2max_ 54.8 (5.6) ml/kg BM/min], volunteered to participate in the study. All participants gave written informed consent. The study protocol received approval from the Monash University Human Research Ethics Committee (MUHREC: 12799) and conformed with the 2008 Helsinki Declaration for Human Research Ethics. Standard exclusion criteria were applied as previously described (Costa et al., 2017a). Data presented within is additional follow-on data analysis from previous original experimental research (Russo et al., 2021a; Russo et al., 2021b; Russo et al., 2021c), registered with the Australian and New Zealand Clinical Trials Register (ANZCTR reference number 375090).

### Preliminary measures

One to 3 weeks prior to the first experiment trial, baseline measurements for height (Stadiometer, Holtain Limited, Crosswell, Crymuch, United Kingdom), body mass (BM) (Seca 515 MBCA, Seca Group, Hamburg, Germany), body composition (Seca 515 MBCA, Seca Group, Hamburg, Germany) and *V̇*O_2max_ (Vmax Encore Metabolic Cart, Carefusion, San Diego, CA, United States) were recorded. *V*O_2max_ was estimated by means of a continuous incremental exercise test to volitional exhaustion on a motorized treadmill (Forma Run 500, Technogym, Seattle, WA, United States), as previously reported (Costa et al., 2009). Criteria for attaining *V̇*O_2max_ included participant reaching volitational exhaustion [i.e., rating of perceived exertion (RPE) of 19-20 Borg scale], a heart rate (HR) within 10 beats/min of HR_max_, with observation of *V̇*O_2_ plateau in increasing exercise intensity and/or inclusion of RER (1.100). To determine running speeds for the exercise trials, the speed at approximately 50 [mean (SD): 7.3 (1.0) km/h], 55-60 [8.7 (1.3) km/h], 70-75 [10.8 (1.4) km/h], and 80-85 [12.7 (1.8) km/h] % *V̇*O_2max_ and 1% gradient was determined and verified from the *V̇*O_2_-work rate relationship.

### Experimental procedures

**FIGURE 1:**
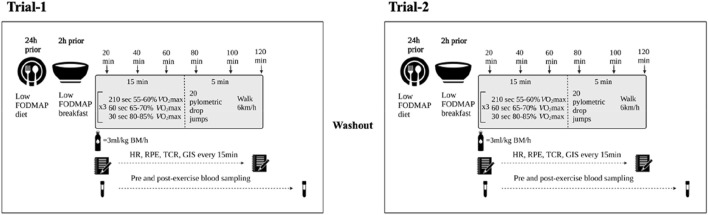
Schematic illustration of experimental procedures.

A schematic illustration of the experimental procedures is depicted in Figure 1. Participants undertook two exertional-stress experimental protocols. To accommodate participant availability, washout ranged from 5-days to 14-days washout (mean 10-days). Considering the potential impact of dietary intake on the measured gastrointestinal variable in response to exercise (Costa et al., 2016), participants were provided with a standard low FODMAP diet the day before the experimental trials [energy 10.1 (3.0) MJ/day, protein 98 (30) g/day, fat 57 (36) g/day, carbohydrate 353 (87) g/day, fiber 44 (11) g/day, water 2.3 (1.4) L/day, <5 g/day FODMAP]. Dietary compliance was assessed using a food-fluid dietary log and compliance checklist. In addition, participants were asked to avoid alcohol and strenuous exercise for 48 h prior, and avoid consumption of caffeinated beverages 24 h prior to each experimental trial. Trials for female athletes were scheduled during the follicular phase of their menstrual cycle or when taking the active medication of oral contraceptive pill. Resting estrogen levels (DKO003/RUO; DiaMetra, Italy) were measured for verification and were within normal range for both trials (<20.0 pg/ml).

On the day of experimental trials, the participants reported to the laboratory at 0800 h after consumption of a standardised low FODMAP breakfast [energy 2.9 (0.8) MJ, protein 28 (9) g, fat 19 (5) g, carbohydrate 99 (28) g, fiber 12 (5) g, and water 363 (264) ml] at 0700 h. From arrival to the lab until the initiation of exercise, participants were asked to void, and in addition, provide a 30 g mid-flow fecal sample (n = 27) collected in a sterilised fecal collection container (SARSTEDT Australia Pty Ltd, Mawson Lakes, South Australia, Australia). Fecal samples were immediately stored frozen at −80°C until sample processing and analysis. Nude BM and total body water (TBW) (Seca 515 MBCA, Seca Group, Hamburg, Germany) were then recorded prior to exercise. A breath sample was taken using 250 ml collection bag (Wagner Analysen Tecknik, Bremen, Germany) and blood samples collected by venepuncture from an antecubital vein into two separate vacutainers (6 ml 1.5 IU/ml lithium heparin and 4 ml 1.6 mg/ml K_3_EDTA; BD, Oxford, United Kingdom). Participants completed an exercise-specific modified visual analog scale (mVAS) GIS assessment tool (Gaskell et al., 2019) and prior to starting exercise, inserted a thermocouple 12 cm beyond the external anal sphincter to record pre-exercise rectal temperature (T_re_) (Precision Temperature 4600 Thermometer, Alpha Technics, CA, United States).

The exercise protocol began at 0900 h and consisted of 2 h high intensity interval (HIIT) running in T_amb_ 23.0 (1.0)°C and 43.5 (7.5)% relative humidity (RH) (Figure 1). Participants completed 6 × 20-min circuits consisting of 3 × 5-min of running at varying intensities (210 s at 50%–60% *V̇*O_2max_, 60 s at 65%–70% *V̇*O_2max_ and 30 s at 80%–85% *V̇*O_2max_) for a total of 15-min followed by 20 plyometric drop jumps and then walking at 6 km/h for the remaining 5-min of the 20-min cycle. The protocol was established based on exercise stress models that perturb immune and gastrointestinal status compared with baseline (Costa et al., 2009; Costa et al., 2011; Snipe and Costa, 2018a; Snipe and Costa, 2018b; Costa et al., 2019; Russo et al., 2019; Costa R. J. S. et al., 2020). Participants were provided with water equivalent to 3 ml/kgBM/h during exercise (Costa et al., 2009, Costa et al., 2011). Measures of HR (Polar Electro, Kempele, Finland), RPE and McGinnis 13-point thermal comfort rating scale (TCR) were taken during exercise at the 15-min mark of every 20-min cycle (Costa et al., 2014), and HR and Ex-GIS were measured during the final 30 s of a 20-min cycle. Immediately post-exercise, nude BM and T_re_ were recorded. Blood samples were then collected 30 min into recovery as part of the primary recovery intervention research (Russo et al., 2021a; Russo et al., 2021b; Russo et al., 2021c). Breath samples and GIS were recorded every 30 min throughout the 2 h recovery period.

### Blood sample analysis

The HemoCue system (Glucose 201+, Hb201, and WBC DIFF, HemoCue AB, Angelholm, Sweden) was used to determine blood glucose concentration, haemoglobin, total and differential leukocyte counts (including neutrophils, lymphocytes and monocytes) in duplicate from whole blood samples. The coefficient of variation (CV) for blood glucose concentration, haemoglobin and leukocyte counts was 5.1%, 1.6%, and 13.6% respectively. Haematocrit was determined by capillary method in triplicate from heparin whole blood samples using a microhematocrit reader (CV: 0.7%) (Thermo Fisher Scientific). The haemoglobin and haematocrit values were used to determine changes in plasma volume (P_v_) relative to baseline, and to correct plasma variables (Dill and Costill, 1974). Bacterially-stimulated elastase release was determined as previously described (Costa et al., 2009; Costa et al., 2011; Costa et al., 2019a; Costa R. J. S. et al., 2020). The remaining whole blood in the heparin and K3EDTA vacutainers was centrifuged at 4,000 rpm (1,500 g) for 10 min within 15 min of sample collection and aspirated into 1.5 ml micro-storage tubes and frozen at −80°C until analysis. Prior to freezing, two 50 µl aliquots of heparin plasma were used to determine plasma osmolality (P_Osmol_), in duplicate (CV: 0.7%), by freeze point osmometry (Osmomat 030, Gonotec, Berlin, Germany). Circulating concentrations of cortisol (DKO001; DiaMetra, Italy), PMN elastase (BMS269; Affymetrix EBioscience, Vienna, Austria), I-FABP (HK406; Hycult Biotech, Uden, Netherlands), sCD14 (HK320; Hycult Biotech), and LBP (HK315, Hycult Biotech) were determined by ELISA. Additionally, systemic cytokine profile [i.e., plasma IL-1β, TNF-α, IL-10, and IL-1ra concentrations] (HCYTMAG-60K, EMD Millipore, Darmstadt, Germany) were determined by multiplex system (Magpix, Luminex, Austin, TX, United States). All variables were analysed as per manufacturer’s instructions on the same day, with standards and controls on each plate, and sample from each participant assayed on the same plate. The intra- and inter-assay CV for analysed biomarkers, respectively, was 6.1% and 10.4% for cortisol, 2.8% and 3.6% for I-FABP, 4.0% and 9.3% for LBP, 3.3% and 4.2% for sCD14, 5.5% and 9.7% for elastase, 16.0% and 16.6% for IL-1β, 14.9% and 15.5% for TNFα, 15.8% and 9.1% for IL-6, 14.7% and 12.6% for IL-8, 15.9% and 11.1% for IL-10, and 9.2% and 8.8% for IL-1ra.

### Fecal and plasma microbial profiling

Upon thawing at room temperature, fecal samples were homogenised, then 0.20–0.30 g of each sample were transferred to a 2 ml dry garnet bead microtube, before the addition of bead solution. Cell lysis, sample purification, and DNA extraction was then performed as per manufacturer’s instructions (PowerFecal DNA isolation kit, MoBio Laboratories, Qiagen, Germantown, United States). Blank control samples, using pyrogen/DNAse/RNAse free water in replacement of biological sample, were run simultaneously in duplicate. Purified extracted DNA (50 µl sample) was immediately frozen at −20°C prior to bacterial gene sequencing. Extracted genomic DNA was delivered to the Australian Genome Research Facility (Melbourne, Australia) for PCR amplification of the V3-V4 region of the 16S rRNA gene, and sequencing on the Illumina MiSeq platform utilising the Illumina’s Nextera XT Index kit. Blank control samples yielded undetectable outcomes.

For detection and profiling of bacterial content in plasma samples a QIAamp UPC pathogen mini kit was used to extract microbial DNA (MoBio Laboratories, Qiagen, Germantown, United States), prior to s16 gene sequencing. After thawing, 200 µl of heparin plasma was added to 1.5 ml glass microbead tubes and underwent mechanical lysis in accordance with manufacturer’s instructions. Thereafter, 400 µl of the pre-treated sample underwent the spin protocol for chemical lysis in accordance with the manufacture’s instruction. Purified extracted DNA (100 µl sample) was immediately frozen at −20°C prior to bacterial gene sequencing. Blank control samples, using pyrogen/DNAse/RNAse free water in replacement of biological sample, were run simultaneously in duplicate. Extracted genomic DNA was delivered to the Micromon Next-Generation Sequencing Facility (Monash University, Clayton, Australia) for PCR amplification of the V3-V4 region of the 16S rRNA gene as previously described (Bennett et al., 2020). Prior to s16 gene sequencing, microbial DNA detection and concentration was performed by Qubit fluorometer quantification (ThermoFisher Scientific, Waltham, MA, United States) in duplicate (CV: 2.5%). Blank control samples yielded undetectable outcomes.

The assembled reads were analysed using QIIME2 (v.2019.1), as previously described (Bennett et al., 2020). Briefly, reads were imported into QIIME2 with quality assessment, filtering, barcode trimming, and chimera detection were performed using the DADA2 pipeline. Taxonomic evaluation using pre-set parameters (98% identify, confidence *p* <0.05%) with the SILVA 138.1 release (Quast et al., 2013). Sequencing data are available on the Short Read Archive (SRA, https://www.ncbi.nlm.nih.gov/sra), BioProject number PRJNA926792. Before statistical analysis, sequencing data for phyla, family, and genus amplicon sequence variants were calculated by dividing the number of reads for each taxon by the number of reads in the fecal and plasma samples.16S rRNA sequences per fecal and plasma samples ranged from 33,197 to 219,089 and 13,091 to 42,715, respectively. To minimise the risk of including artefact values in data analysis, resulting from potential contamination during sample handling (i.e., sample collection, processing and analysis procedures), a fresh sample was collected and immediately processed and frozen. The samples were processed in a sterile laboratory and UV biological grade fume cabinet (Safemate 1.2 ECO, LAF Technologies Pty Ltd, Baywater North, Victoria, Australia) and pyrogen/DNAse/RNAse free sample processing consumables were used. For amplicon sequence variants (AVS), only bacterial groups with a conservative ≥0.5% relative abundance, respective to the determination medium, were included for data analysis (Nikkari et al., 2001; Luo et al., 2021).

Bacterial calculations of n = 5 and n = 5 phyla, n = 22 and n = 28 family, n = 40 and n = 22 genus AVS, for fecal and plasma samples respectively, were adequately detected for relative abundance and α-diversity (i.e., Shannon Index (SI) and Shannon Equitability Index (SEI) determination.

### Fecal and plasma short chain fatty acid (SCFA) analysis

Fecal SCFAs were measured by gas chromatography as previously described (Clarke et al., 2011). Thawed fecal material was spiked with three times the volume of internal standard (1.68 mM heptanoic acid), homogenized and centrifuged (2000 g, 10 min, 4°C). After centrifugation, 300 μl of supernatant was added to a 0.2 μm filter vial containing 10 μl of 1 M phosphoric acid. The vials were then analysed for SCFA content *via* gas chromatography. Samples were analysed using an Agilent GC6890 gas chromatography coupled to a flame-ionisation detector (FID), with helium used as the carrier gas. An Agilent free fatty acid phase (FFAP) column (30 m × 0.53 mM (internal diameter) x 1.00 μM (film thickness) was installed for analysis. A splitless injection technique was used, with 0.2 μl of sample injected. A constant flow rate of 4.0 ml/min was used on the column. Upon injection, the oven was initially held at 90°C for 1 min, then raised to 190°C at 20°C/min and held for 3 min. Samples were run in triplicate to ensure accurate and replicable data were obtained. A CV <10% within triplicate samples was used as a quality control measure.

Plasma samples (heparin) were analysed in duplicate for SCFA content using gas-chromatography, as previously described (Gill et al., 2020). Briefly, 300 µl of plasma was spiked with 50 µl of 200 µM heptanoic acid and acidified with the addition of 50 µl of 10% sulfosalicylic acid before the addition of 3 ml diethyl ether solvent. The mixture was vortexed and centrifuged so that the organic layer could be clarified and transferred into 50 µl 0.2 M NaOH. The alkaline solution containing SCFA was concentrated by evaporation using nitrogen, dissolved in 30 µl 1 M phosphoric acid and transferred into a cold GC glass vial for analysis using an Agilent GC6890 coupled to FID. Concentrations for acetate, propionate and butyrate were determined by the average of the triplicate results, where the CV was <20%. Total SCFA was calculated by the sum of the individual SCFA. Results were expressed as µmol/L.

### Statistical analysis

Of the original metadata collection (Russo et al., 2021a; Russo et al., 2021b; Russo et al., 2021c), confirmation of adequate statistical power was determined *a priori* for EIGS biomarkers (i.e., circulating leukocytes, and plasma concentration of I-FABP, bacterial endotoxins, and inflammatory cytokines) and Ex-GIS by applying the mean, standard deviation, and effect size on these variable in response to exertional stress (Costa et al., 2009; Costa et al., 2011; Costa et al., 2017a; Snipe and Costa, 2018a; Snipe and Costa, 2018b; Costa et al., 2019; Costa R. J. S. et al., 2020). Using G Power software, and applying a standard alpha (0.05) and beta value (0.80), the current participant sample size is estimated to provide adequate statistical power (power* 0.80-0.99) for detecting if there are any significant differences between the test-to-retest EIGS biomarker values (G_Power 3.1, Kiel, Germany), which was confirmed using *post hoc* software test application. Statistics were analysed using SPSS statistical software (V.27.0, IBM SPSS Statistics, IBM Corp., Armonk, NY, United States) with significance accepted at *p* ≤ 0.05. Data in text and tables are presented as mean and 95% confidence interval (CI) for variables. Intraclass correlation coefficient (ICC) was used for test-retest reliability analysis whereby ICC (absolute agreement, 2-way random effects model) range *r* ^*a*^ < 0.50, *r* ^*a*^ *=* 0.50-0.74, *r* ^*a*^ *=* 0.75-0.90, and *r* ^*a*^ > 0.90 was interpreted as poor, moderate, good and excellent, respectively (Koo & Li, 2016). Standard error of mean (SEM) was calculated using established formula: $SEM=SD\times \sqrt{1}-ICC$ and minimal detectable change (MDC) was calculated using previously established formula: MDC=SEM×1.96×√2 (Weir, 2005). Significance of test-retest variation was determined by the Wilcoxon signed-rank test. Magnitude of test-retest variation was determined by Cohen’s *d* standardized measurement of effect size, whereby *d* < .20, *d* = .20 to .49, *d* = .50 to .80, and *d* > .80 for no, small, medium, and large effects, respectively. Test-retest correlation and agreement were assessed using Bland–Altman plots calculating bias and limits of agreement, as well as Spearman’s correlation coefficients (whereby r_s_< 0.300, r_s_ = 0.300 to 0.500, and r_s_> 0.500 for weak, moderate, and strong, respectively). Limits of agreement were defined as mean bias ± 2 SD.

## Results

### Physiological strain

**FIGURE 2:**
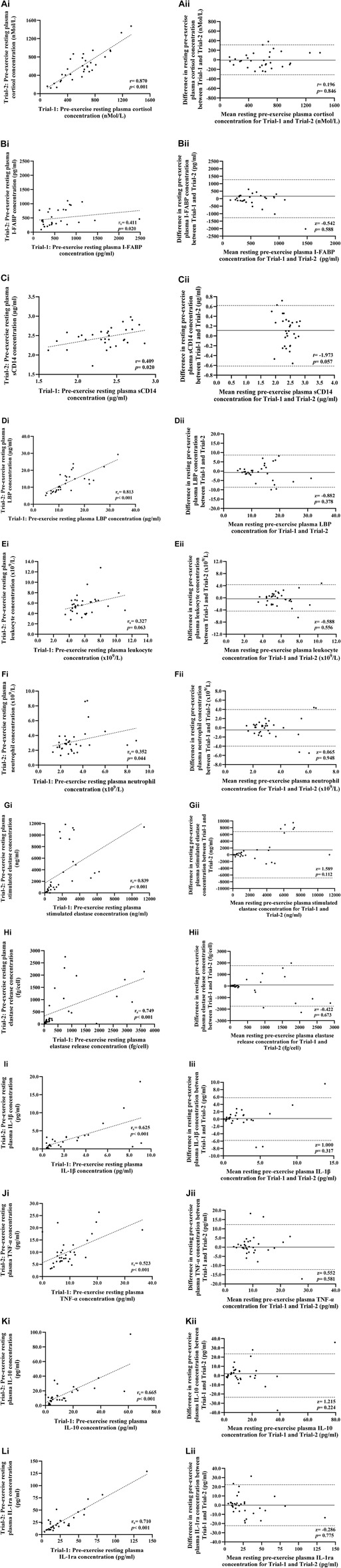
Correlation i) and Bland-Altman plot ii) comparing combined data from Trial-1 and Trial-2 for resting pre-exercise plasma cortisol **(A)**, I-FABP **(B)**, sCD14 **(C)**, and LBP **(D)** concentration; total leukocyte **(E)** and neutrophil counts **(F)**; total bacterially-stimulated elastase release **(G)** and elastase release per cell **(H)**; and plasma IL-1β **(I)**, TNF-α **(J)**, IL-10 **(K)**, and IL-1ra **(L)** concentration. Dotted line represents limits of agreement (±2 SD; 95% confidence interval), and the solid line represents mean bias between trials.

The indices of physiological strain measured on Trial-1 and Trial-2 are reported in Table 1. Pre- and post-exercise P_Osmol_ was 291 (288–294) and 293 (290–296) mOsmol/kg, respectively, and was not significantly different between trials (*p* = 0.052 and *p* = 0.546, respectively). Exercise-induced body mass loss and Pv change was 1.9 (1.7–2.1) % and −2.1 (−3.8 to −0.6) %, respectively, and was not significantly different between trials (*p* = 0.485 and *p* = 0.998, respectively). No difference in resting pre-exercise and post-exercise plasma cortisol concentration was observed between trials (Table 2). However, a significant increase in pre-to post-exercise plasma cortisol concentration was observed on Trial-1 only, but no substantial difference in magnitude of response was observed between the two trials (Table 3). Assessment of test-retest presented a strong correlation for resting pre-exercise plasma cortisol concentration, with good test-retest reliability (Figure 2A; Table 4), and a moderate correlation (*r* = 0.455) with poor test-retest reliability (ICC = 0.41) for exercise-associated change in plasma cortisol concentration (Supplementary Table S1; Figure 1).

**TABLE 1:** Indices of physiological strain measured during Trial-1 and Trial-2 in response to 2 h high intensity interval exercise in temperate ambient conditions.

	**Trial-1**	**Trial-2**	**p**
Mean heart rate- low pace (bpm)	125 (121–130)	120 (116–125)	**<.001**
Mean heart rate- high pace (bpm)	160 (156–165)	157 (152–162)	**.005**
Cardiac drift—low pace (bpm)	14 (11–16)	14 (11–17)	.750
Cardiac drift—high pace (bpm)	10 (8–12)	9 (7–10)	.214
Mean rating of perceived exertion	13 (13–14)	12 (12–13)	**<.001**
Δ rating of perceived exertion^a^	3 (3–4)	3 (3–4)	.781
Post-exercise T_re_ (°C)	37.9 (37.6–38.2)	37.8 (37.6–38.1)	.810
Δ T_re_ (°C)^b^	1.2 (0.9–1.6)	1.2 (0.9–1.4)	.795
Mean thermal comfort rating	9 (8–9)	9 (8–9)	.283
Δ thermal comfort rating^a^	2 (1–2)	1 (1–1)	.146

Mean and 95% CI (n = 34).

^a^ Change from cycle 1 to cycle 6, and pre-to post-exercise change.

**TABLE 2:** Pre- and post-exercise exercise-induced gastrointestinal syndrome (EIGS) biomarkers in response to 2 h high intensity interval exercise in temperate ambient conditions performed on two separate occasions.

	**Pre Trial-1**	**Pre Trial-2**	*d*	**Post Trial-1**	**Post Trial-2**	*d*
Cortisol (nMol/L)	603 (502–705)	598 (487–709)	.00	831 (692–970)**	716 (566–866)	.23
IFABP (pg/ml)	614 (444–783)	498 (400–596)	.22	1275 (940–1610)**	1136 (865–1406)**	.13
sCD14 (µg/ml)	2.4 (2.2–2.5)	2.5 (2.4–2.5)	.30	2.3 (2.2–2.4)	2.5 (2.3–2.6)	.40
LBP (µg/ml)	13.5 (11.2–15.8)	12.9 (10.8–15.0)	.08	14.4 (12.0–16.8)	13.4 (11.0–15.8)	.13
Total leukocyte counts (x10^9^/L)	5.9 (5.3–6.5)	5.7 (5.0–6.4)	.11	8.0 (7.1–8.9)**	7.2 (6.5–7.9)**	.29
Neutrophil counts (x10^9^/L)	3.3 (2.7–3.8)	3.2 (2.6–3.8)	.03	5.1 (4.3–5.9)**	4.4 (3.8–4.9)**	.27
Total stimulated elastase (ng/ml)	2217 (1351–3083)	3745 (2262–5227)	.40	3739 (2402–5076)**	4938 (3382–6494)**	.25
Elastase release per cell (fg/ml)	749 (379–1119)	650 (363–937)	.08	350 (236–464)	588 (363–836)	.44
IL-1β (pg/ml)	2.2 (1.2–3.1)	2.4 (1.2–3.7)	.07	2.0 (1.2–2.8)	2.6 (1.4–3.8)	.17
TNF-α (pg/ml)	9.6 (7.3–12.0)	10.3 (8.3–12.3)	.08	10.8 (8.4–13.1)	10.5 (8.4–12.3)	.03
IL-10 (pg/ml)	13.1 (7.9–18.3)	15.2 (9.2–21.2)	.12	27.4 (18.7–36.2)**	23.0 (16.7–29.3)**	.15
IL-1ra (pg/ml)	28.5 (18.9–38.1)	28.5 (19.8–37.2)	.00	33.7 (25.1–42.4)**	35.9 (26.0–45.7)**	.07
Plasma bacterial DNA (ng/µl)	0.03 (0.02–0.05)	0.03 (0.01–0.05)	.17	0.07 (0.06–0.08)**	0.07 (0.06–0.08)**	.07
Plasma phyla SEI	0.245 (0.213–0.276)	0.275 (0.252–0.294)	.44	0.217 (0.195–0.240)**	0.214 (0.195–0.233)**	.07
Plasma family SEI	0.292 (0.276–0.308)	0.295 (0.278–0.312)	.10	0.292 (0.278–0.305)	0.300 (0.284–0.316)	.25
Plasma genus SEI	0.244 (0.224–0.265)	0.242 (0.226–0.259)	.05	0.253 (0.239–0.267)	0.271 (0.260–0.282)**	.62

Mean and 95% CI. ** *p* < 0.001 *vs*. pre-exercise. Magnitude of test-retest variation was determined by Cohen’s *d* standardized measurement of effect size, whereby *d* < .20, *d* = .20 to .49, *d* = .50 to .80, and *d* > .80 for no, small, medium, and large effects, respectively.

**TABLE 3:** Pre-to post-exercise magnitude of change in exercise-induced gastrointestinal syndrome (EIGS) biomarkers in response to 2 h high intensity interval exercise in temperate ambient conditions performed on two separate occasions.

	**Δ Trial-1**	**Δ Trial-2**	*d*
Cortisol (nMol/L)	228 (99–357)	118 (−24–260)	.23
IFABP (pg/ml)	661 (357–964)	638 (390–885)	.02
sCD14 (µg/ml)	−0.02 (−0.12 to 0.08)	0.04 (−0.10–0.18)	.14
LBP (µg/ml)	0.91 (−0.20–2.00)	0.47 (−0.74–1.70)	.11
Total leukocyte counts (x10^9^/L)	2.1 (1.3–2.9)	1.5 (0.6–2.3)	.22
Neutrophil counts (x10^9^/L)	1.8 (1.1–2.6)	1.2 (0.6–1.8)	.26
Total stimulated elastase (ng/ml)	1522 (761–2283)	1193 (104–2283)	.12
Elastase release per cell (fg/ml)	−398 (−756 to −41)	−51 (−288 to 187)	.31
IL-1β (pg/ml)	−0.2 (−0.5 to 0.2)	0.2 (−0.5–0.8)	.18
TNF-α (pg/ml)	1.1 (−0.1–2.3)	0.2 (−1.2–1.6)	.21
IL-10 (pg/ml)	14.3 (6.6–22.0)	7.8 (3.8–11.8)^a^	.27
IL-1ra (pg/ml)	5.3 (0.7–9.8)	7.4 (4.0–10.7)	.14
Plasma bacterial DNA (ng/µl)	0.04 (0.02–0.05)	0.04 (0.02–0.07)	.19
Plasma phyla SEI	−0.03 (−0.06 to 0.00)	−0.06 (−0.09 to −0.03)	.41
Plasma family SEI	0.00 (−0.01 to 0.01)	0.00 (−0.02–0.03)	.13
Plasma genus SEI	0.01 (−0.01–0.03)	0.03 (0.02–0.04)	.46

Mean and 95% CI.

^a^ *p* < 0.05 magnitude of response Trial-1 *vs*. Trial-2. Magnitude of test–retest variation was determined by Cohen’s *d* standardized measurement of effect size, whereby *d* < .20, *d* = .20 to .49, *d* = .50 to .80, and *d* > .80 for no, small, medium, and large effects, respectively.

**TABLE 4:** Reliability indices of pre-exercise resting exercise-induced gastrointestinal syndrome (EIGS) biomarkers (A), plus fecal and plasma microbial α-diversity (B) and short chain fatty acid (SCFA) concentrations (C) between Trial-1 and Trial-2.

	**Intraclass correlation coefficient r** **^a^**	**95% CI**	**p**	**SEM** **^b^**	**MDC** **^c^**
** A. EIGS biomarkers**					
Cortisol (nMol/L)	0.87	0.75 to 0.93	**<.001**	134	370
IFABP (pg/ml)	0.21	−0.14 to 0.51	.199	469	1301
sCD14 (µg/ml)	0.38	0.06 to 0.64	**.010**	0.28	0.78
LBP (µg/ml)	0.76	0.56 to 0.88	**<.001**	3.9	10.7
Total leukocyte counts (x10^9^/L)	0.33	−0.02 to 0.60	**.032**	1.83	5.07
Neutrophil counts (x10^9^/L)	0.32	−0.04 to 0.59	**.038**	1.71	4.74
Total stimulated elastase (ng/ml)	0.44	0.12 to 0.68	**.004**	2843	7882
Elastase release per cell (fg/ml)	0.54	0.22 to 0.75	**.001**	800	2218
IL-1β (pg/ml)	0.64	0.39 to 0.81	**<.001**	2.28	6.31
TNF-α (pg/ml)	0.56	0.27 to 0.75	**<.001**	5.3	14.6
IL-10 (pg/ml)	0.73	0.53 to 0.86	**<.001**	10.2	28.2
IL-1ra (pg/ml)	0.92	0.84 to 0.96	**<.001**	9.5	26.2
Plasma bacterial DNA (ng/µl)	0.47	−0.03 to 0.79	**.035**	0.03	0.08
**B. Microbial α-diversity**					
**Fecal SEI** ^d^					
Phyla	0.29	−0.17 to 0.67	.114	0.07	0.18
Family	0.56	0.08 to 0.83	**.014**	0.03	0.07
Genus	0.54	0.04 to 0.82	**.019**	0.03	0.08
**Plasma SEI** ^d^					
Phyla	0.49	0.03 to 0.79	**.012**	0.05	0.13
Family	0.04	−0.52 to 0.54	.448	0.03	0.09
Genus	−0.16	−0.67 to 0.39	.712	0.04	0.04
**C. SCFA**					
Total fecal SCFA (µg/g)	0.53	0.17 to 0.77	**.003**	35.3	97.7
Fecal acetate (µg/g)	0.52	0.16 to 0.77	**.003**	26.6	73.7
Fecal propionate (µg/g)	0.44	0.05 to 0.72	**.016**	6.8	19.0
Fecal butyrate (µg/g)	0.45	0.04 to 0.73	**.017**	6.3	17.4
Fecal valeric (µg/g)	0.48	0.08 to 0.75	**.012**	1.1	2.9
Fecal caproic (µg/g)	0.56	0.20 to 0.79	**.003**	0.6	1.7
Total plasma SCFA (µg/L)	0.59	0.29 to 0.78	**<.001**	62.3	172.8
Plasma acetate (µg/L)	0.59	0.28 to 0.79	**<.001**	60.5	167.6
Plasma propionate (µg/L)	0.14	−0.02 to 0.46	.212	6.2	17.3
Plasma butyrate (µg/L)	0.63	0.37 to 0.79	**<.001**	2.9	7.9

^a^ Intraclass correlation coefficient (ICC), whereby *r* ^*a*^ < 0.5, *r* ^*a*^ *=* 0.5-0.74, *r* ^*a*^ *=* 0.75-0.9, and *r* ^*a*^ > 0.90 for poor, moderate, good and excellent reliability respectively and 95% confidence intervals (CI).

^b^ SEM (Standard Error of Measurement).

^c^ MDC (Minimal Detectable Change).

^d^ SEI (Shannon Equitability Index).

### Intestinal integrity

No difference in resting pre-exercise and post-exercise plasma I-FABP, sCD14 or LBP concentration was observed between trials (Table 2). Plasma I-FABP concentration increased in response to HIIT in Trial-1 and Trial-2, whereas plasma sCD14 and LBP concentration did not significantly change in either trial (Table 2). The magnitude of response for these biomarkers did not significantly differ between trials (Table 3). Assessment of test–retest presented a strong correlation with good test-retest reliability for resting pre-exercise plasma LBP concentration, and a moderate correlation with poor test-retest reliability for plasma I-FABP and sCD14 (Figures 2B–D, respectively; Table 4). The correlation for exercise-associated change between trials was moderate for plasma I-FABP concentration, and weak for plasma sCD14 concentration and LBP concentration (*r* = 0.319, *r* = −0.079, and *r* = 0.119, respectively) while test-retest reliability was poor for all three biomarkers (ICC ≤0.21) (Supplementary Table S1; Figure 1).

### Leukocyte trafficking and bacterially-stimulated neutrophil degranulation

No difference in resting pre- and post-exercise total leukocyte and neutrophil counts were observed between trials (Table 2). Total leukocyte and neutrophil counts increased in response to HIIT in both Trial-1 and Trial-2 (Table 2). No difference in magnitude of response was observed between the two trials for these blood cell counts (Table 3). Assessment of test-retest correlation for resting total leukocyte and neutrophil counts was moderate with poor test-retest reliability (Figures 2E, F; Table 4), while exercise-associated change in total leukocyte and neutrophil counts presented moderate correlations (*r* = 0.487 and *r* = 0.468, respectively) with poor test-retest reliability (ICC ≤0.48) (Supplementary Table S1; Figure 1).

There was no difference in resting pre- and post-exercise total bacterially-stimulated elastase and elastase release per cell between trials (Table 2). The concentration of total bacterially-stimulated elastase increased in response to HIIT in both Trial-1 and Trial-2. No significant change was observed in elastase release per cell in either trial (Table 2). The magnitude of response for these *in-vitro* immune function biomarkers did not significantly differ between trials (Table 3). Assessment of test-retest revealed a strong correlation for resting plasma total bacterially-stimulated elastase (Figure 2G), but poor-to-moderate test-retest reliability (Table 4). Exercise-associated change from pre-to post-exercise for plasma total bacterially-stimulated elastase presented a strong correlation (*r* = 0.646), with good test-retest reliability (ICC = 0.72). A strong pre-exercise correlation, but moderate test-retest reliability (Figure 2H; Table 4), was presented for plasma elastase release per cell, with no correlation (*r* = 0.073) and poor test-retest reliability observed for exercise-associated change in elastase release per cell (ICC = 0.23) (Supplementary Table S1; Figure 1).

### Systemic inflammatory cytokine profile

No difference in resting pre- and post-exercise plasma concentrations of IL-1β, TNF-α, IL-10 or IL-1ra was observed between trials (Table 2). An increase in plasma concentration pre-to post-exercise of anti-inflammatory cytokines, IL-10 and IL-1ra, was observed in both Trial-1 and Trial-2, while no other cytokines significantly changed in response to exercise in either trial (Table 2). A difference in magnitude of response of plasma IL-10 concentration was observed between Trial-1 and Trial-2 (Table 3). No substantial difference in magnitude of response was observed for any other systemic inflammatory cytokines. Assessment of test-retest presented a strong correlation for resting concentrations of all measured systemic inflammatory cytokines (Figures 2I–L). Test-retest reliability of resting plasma cytokine concentrations presented as excellent for IL-1ra, good for IL10, and moderate for IL-1β and TNF-α (Table 4). Weak correlations were observed between exercise-associated change for all systemic inflammatory cytokines, with the exception of plasma IL-10 concentration, which demonstrated a strong correlation between Trial-1 and Trial-2 (*r* = 0.615). All cytokines presented poor test-retest reliability for exercise-associated change in systemic inflammatory cytokine concentration (ICC ≤0.48) (Supplementary Table S1; Figure 1).

### Pre-exercise fecal bacteria composition

At rest, the sufficient identification of relative abundance of bacterial phyla groups in fecal samples included: *Firmicutes* (69%), *Bacteroidota* (24%), *Actinobacteroita*, *Proteobacteria,* and *Verrucomicrobia* (2%), which did not substantially differ between trials (*p* > 0.05, *d* ≤ .46). Resting fecal bacterial phyla SEI was 0.188 (95% CI: 0.166 to 0.211) and did not substantially differ between trials (*p* = 0.098, *d* = .42); but there was no significant Trial-1 *vs*. Trial-2 correlation observed (r = 0.325, *p* = 0.237) (Table 4).

At rest, the sufficient identification of relative abundance of bacterial family groups in fecal samples included: *Ruminococcaceae* (27%), *Lachnospiraceae* (27%), *Bacteriodaceae* (13%), *Acidaminococcaceae* (6%), *Prevotellaceae* (5%), *Christensenellaceae* (4%), *Veillonellaceae* (3%), *Rikenellaceae*, *Muribaculaceae*, *Akkermensiaceae*, *Pasteurellaceae*, and *Bifidobacteriaceae* (2%), and all other identified bacterial family groups (n = 10) at ≤1%. The relative abundance of bacterial family groups at rest did not substantially differ between trials (*p* > 0.05, *d* ≤ .47), except for *Akkermensiaceae* (Trial-1 3.1% and Trial-2 0.6%; *p* = 0.021, *d* = .46). Resting fecal bacterial family SEI was 0.245 (95% CI: 0.234 to 0.256) and did not substantially differ between trials (*p* = 0.440, *d* = .16), with a significant Trial-1 *vs*. Trial-2 correlation observed (r = 0.554, *p* = 0.032) (Table 4).

At rest, the sufficient identification of relative abundance of bacterial genus groups in fecal samples included: *Bacteroides* (13%), *Faecalibacterium* (11%), *Agathobacter* (5.7%), *Phascolarctobacterium* (5.3%), *Prevotella 9* (4.3%), *Blautia* (4.2%), *Christensenellaceae R-7 group* (3.6%), *Roseburia* (3.5%), *Subdoligranulum* (3.2%), *Alistipes* (2.4%), *Veillonella* (2.1%), and *Eubacterium-coprostanoligenes group* (2.0%), and all other identified bacterial genus groups (n = 28) at ≤2%. The relative abundance of bacterial genus groups at rest did not significantly differ in the larger majority of detectable genera (*p* > 0.05, *d* ≤ .48). Differences between Trial-1 and Trial-2 were only observed for *Akkermensia* (Trial-1 3.1% and Trial-2 at 0.6%; *p* = 0.021, *d* = .46), *Coprococcus-3* (Trial-1 0.5% and Trial-2 at 0.9%; *p* = 0.045, *d* = .56), *Dorea* (Trial-1 0.7% and Trial-2 at 1.3%; *p* = 0.036, *d* = .73), *Parabactercides* (Trial-1 0.9% and Trial-2 at 0.6%; *p* = 0.040, *d* = .27), and *Romboutsia* (Trial-1 0.3% and Trial-2 at 1.6%; *p* = 0.046, *d* = .47). Resting fecal bacterial genus SEI was 0.282 (95% CI: 0.269 to 0.296) and did not substantially differ between trials (*p* = 0.632, *d* = .11), with a significant Trial-1 *vs*. Trial-2 correlation observed (r = 0.585, *p* = 0.022) (Table 4).

### Pre- and post-exercise plasma bacteria composition

Plasma bacterial DNA concentrations significantly increased from pre-to post-exercise in Trial-1 and Trial-2 (*p* < 0.001), with no difference between trials observed (Tables 2, 3). At rest, sufficient identification of relative abundance of bacterial phyla groups in plasma included: *Proteobacteria* (63%), *Firmicutes* (20%), *Actinobacteroita* (7%), *Bacteroidota* and *Cyanobacteria* (4%), which did not substantially differ between trials (*p* > 0.05, *d* ≤ .41), except for *Bacteroidota* (2.8% trial difference; *p* = 0.036, *d* = .65). Resting plasma bacterial phyla SEI did not substantially differ between trials (*p* = 0.032, *d* = .44), with a significant Trial-1 *vs*. Trial-2 correlation observed (r = 0.607, *p* = 0.016). HIIT induced substantial changes in the plasma relative abundance of *Proteobacteria* (+7.9%), *Firmicutes* (−4.2%), and *Cyanobacteria* (−2.9%) (*p* < 0.05). HIIT-induced changes in plasma relative abundance of bacterial phyla did not differ between trials, except for *Bacteroidota* (5.6% trial difference; *p* = 0.005, *d* = .96). These changes translated into a significant reduction in pre-to post-exercise plasma bacterial phyla SEI (*p* < 0.001), which did not differ between trials (Tables 2, 3), and was associated with a correlation trend between Trial-1 *vs*. Trial-2 (r = 0.502, *p* = 0.057) (Table 3).

At rest, sufficient identification of relative abundance of bacterial family groups in plasma included: *Beicojerinckiaceae* (26%), *Halomonadaceae* (10%), *Bacillaceae* (7%), *Pseudomonadaceae* (7%), *Moraxellaceae* (6%), *Erysipelotrichaceae* (5%), *Chloroplast* (4%), *Sphingomonadaceae* (4%), *Lachnospiraceae* (3%), *Corynebacteriaceae*, *Xanthobacteraceae*, *Enterobacteriaceae*, *Comamonadaceae*, *Propionibacteriaceae*, *Chitinophagaceae*, and *Staphylococcaceae* (2%), and all other identified bacterial family groups (n = 12) at ≤1%*,* which did not substantially differ between trials (*p* > 0.05, *d* ≤ 0.75), except for *Bacteroidaceae* (1.7% trial difference; *p* = 0.008, *d* = .79) and *Oxalobacteraceae* (0.6% trial difference; *p* = 0.043, *d* = .54). Resting plasma bacterial family SEI did not substantially differ between trials (*p* = 0.752, *d* = .10) (Table 2). HIIT induced substantial increases in the plasma relative abundance of *Proteobacteria* family groups *Sphingomonadaceae* (+5.8%), *Comamonadaceae* (+2.6%), *Oxalobacteraceae* (+1.2%), *Erwiniaceae* (+1.0%), and *Morganellaceae* (+0.5%); at the expense of reduced *Corynebacteriaceae* (−1.2%), *Bacteroidaceae* (−1.0%), *Chloroplast* (−3.1%), *Lachnospiraceae* (−2.4%), and *Ruminococcaceae* (−0.8%) (*p* < 0.05). These changes did not translate into a significant change in pre-to post-exercise plasma bacterial family SEI (*p* = 0.716), which did not differ between trials (Tables 2, 3).

t rest, sufficient identification of relative abundance of bacterial genus groups in plasma included: *Methylobacterium* (24%), *Halomonas* (10%), *Pseudomonas* (7%), *Anaerobacillus* (5%), *Acinetobacter* (5%), *Erysipelothrix* (5%), *Escherichia-Shigella* (1.9%), *Cutibacterium* (1.6%), *Sandarakinorhabdus* (1.6%), *Corynebacterium* (1.5%), *Bacillus* (1.4%), *Staphylococcus* (1.2%), *Sediminibacterium* (1.2%), and *Bacteroides* (1.2%), and all other identified bacterial genus groups (n = 8) at ≤1%, which did not substantially differ between trials (*p* > 0.05, *d* ≤ .47), except for *Corynebacterium* (1.3% trial difference; *p* = 0.011, *d* = .75) and *Bacteroides* (1.8% trial difference; *p* = 0.006, *d* = .86). Resting plasma bacterial genus SEI did not substantially differ between trials (*p* = 0.878, *d* = .05) (Table 2). HIIT induced substantial increases in the plasma relative abundance of *Proteobacteria* genus groups *Sandarakinorhabdus* (+3.2%), *Sphingomonas* (+1.6%), *Polaromonas* (+1.4%), *Massilia* (+1.3%), *Aquabacterium* (+0.6%), *Providencia* (+0.4%), and increased *Actinobacteriota* genus *Micrococcus* (+0.4%), at the expense of reduced *Bacteroidota* genus *Bacteroides* (−0.9%) (*p* < 0.05). These changes translated into a significant change in pre-to post-exercise plasma bacterial genus SEI (*p* = 0.006), which did not differ between trials (Tables 2, 3).

### Fecal and plasma short-chain fatty acids (SCFA) concentration

**FIGURE 3:**
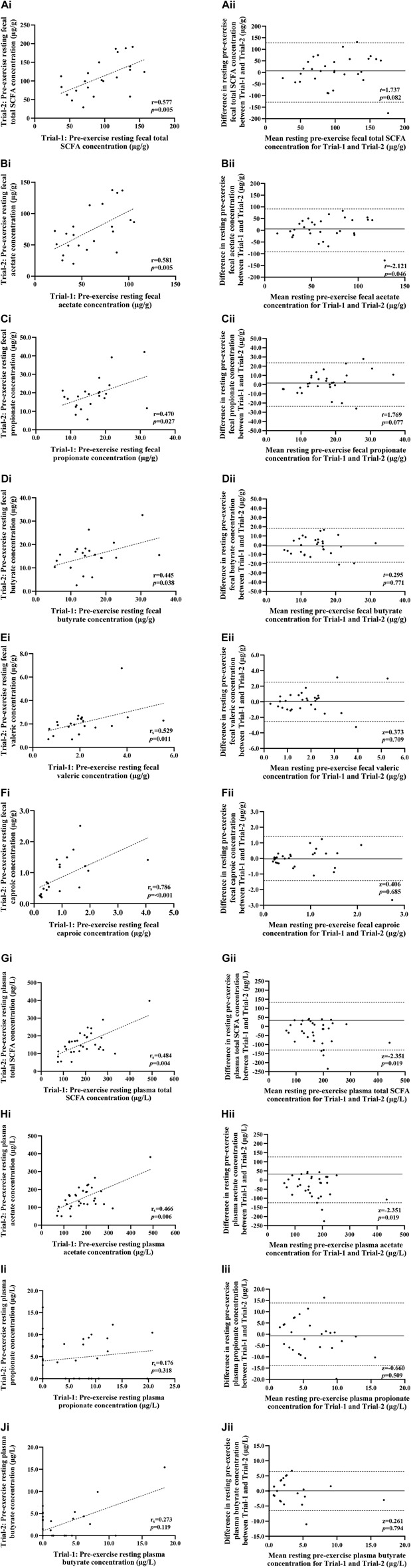
Correlation i) and Bland-Altman plot ii) comparing combined data from Trial-1 and Trial-2 for resting pre-exercise fecal total SCFA **(A)**, fecal acetate **(B)**, fecal propionate **(C)**, fecal butyrate **(D)**, fecal valeric **(E)**, fecal caproic **(F)**, plasma total SCFA **(G)**, plasma acetate **(H)**, plasma propionate **(I)** and plasma butyrate **(J)**. Dotted line represents limits of agreement (±2 SD; 95% confidence interval), and the solid line represents mean bias between trials.

No significant difference was observed between trials in pre-exercise resting total SCFA, acetate, propionate and butyrate fecal and plasma concentrations (Figure 3). Assessment of test-retest presented strong correlations for pre-exercise resting fecal total SCFA, acetate, valeric acid and caproic acid concentrations, and moderate correlations for fecal propionate and butyrate concentrations (Figure 3). Reliability analysis revealed moderate test-retest reliability for fecal measures of total SCFA, acetate and caproic acid concentrations, and poor test-retest reliability for fecal propionate, butyrate, and valeric acid (Table 4). Pre-exercise resting plasma concentrations of total SCFA and acetate concentrations presented moderate correlations (*r* = 0.484 and *r* = 0.466, respectively), while resting pre-exercise plasma propionate and butyrate concentrations presented weak correlations (*r* = 0.176 and *r* = 0.273, respectively). Reliability analysis revealed moderate reliability for pre-exercise total plasma SCFA, acetate, and butyrate concentrations, and poor reliability for pre-exercise plasma propionate (Table 4).

There was no significant relationship between pre-exercise fecal total and differential SCFA concentrations and the magnitude of change in I-FABP concentrations from pre-to post-exercise (*p* > 0.05). A moderate negative correlation was observed between plasma butyrate concentrations and I-FABP (*r* = −0.390 *p* = 0.001), with low levels of resting plasma butyrate concentration associated with increased plasma I-FABP concentrations post-exercise. A weak negative correlation was observed between pre-exercise plasma propionate concentrations and pre-to post-exercise concentrations of plasma IL-1ra (*r* = −0.299, *p* = 0.015) and pre-exercise fecal butyrate concentrations and pre-to post-exercise plasma cortisol (*r* = −0.292, *p* = 0.044).

Moderate positive correlations were observed between pre-exercise fecal total, fecal acetate and fecal caproic concentrations with plasma pro-inflammatory cytokine IL-1β (*r* = 0.413, *p* = 0.004, *r* = 0.472, *p*= <0.001 and *r* = 0.411, *p* = 0.004 respectively). Higher concentrations of fecal total, acetate and caproic pre-exercise were associated with a larger increase of IL-1β from pre-to post-exercise. A moderate positive correlation was observed between pre-exercise fecal acetate concentration and pre-to post-exercise plasma TNF-α concentration (*r* = 0.306, *p* = 0.027). There was no significant relationship between pre-exercise resting fecal and plasma total and differential concentrations of SCFA with the magnitude of change of plasma bacterial DNA from pre-to post-exercise (*p* > 0.05).

## Discussion

The current study primarily aimed to comprehensively determine the test-retest reliability of selected biomarkers linked to EIGS at rest prior to exercise and in response to prolonged strenuous exercise. A secondary aim was to assess the association between luminal and systemic SCFA concentration with these variables. Firstly, at rest, no comparative test differences, with no to low Cohen’s *d* effect size, and significant two-tailed correlation was observed between Trial-1 and Trial-2 for plasma concentrations of cortisol, I-FABP, sCD14, LBP, systemic inflammatory cytokines (i.e., IL-1β, TNF-α, IL-10, and IL-1ra), *in-vitro* bacterially-stimulated elastase, plus neutrophil counts, and fecal and plasma bacterial α-diversity. On the application of ICC, moderate to good test-retest reliability was observed for plasma concentrations of cortisol, LBP, systemic inflammatory cytokines (i.e., IL-1β, TNF-α, IL-10, and IL-1ra), *in-vitro* bacterially-stimulated elastase, and fecal bacterial α-diversity. In response to exercise, 2 h of HIIT perturbed some biomarkers indicative of EIGS, including inducing bacteremia (i.e., quantity and diversity). No comparative test differences, with no to low Cohen’s *d* effect size, and significant (or trend) two-tailed correlation were observed between Trial-1 and Trial-2 in response to exercise for plasma concentrations of cortisol, I-FABP, *in-vitro* bacterially-stimulated elastase, plus leukocyte and neutrophil counts, and plasma bacterial α-diversity. However, on the application of ICC, all markers were considered poor, except for *in-vitro* bacterially-stimulated elastase which was considered moderate for test-retest reliability. Secondly, to the best of our knowledge, this is the first study to directly measure the association between fecal and plasma SCFA concentrations with markers of EIGS. At rest, moderate test-retest reliability was observed for fecal and plasma SCFA concentrations. A novel finding was that pre-exercise plasma butyrate concentration was negatively associated with post-exercise plasma I-FABP concentration, indicative of intestinal epithelial injury. Taken together, the current data indicates: 1) 2 h of HIIT induced mild perturbations to markers indicative of EIGS, including bacteremia; 2) various EIGS biomarkers present good to excellent test-retest reliability at rest in response to rigorous pre-exercise experimental confounder control; 3) no EIGS biomarker presented adequate test-retest reliability in isolation in response to exercise (i.e., 2 h HIIT); and 4) with rigorous experimental control (e.g., standardised dietary provisions), pre-exercise fecal and plasma SCFA concentrations demonstrate an association with biomarkers of EIGS. These outcomes provide support for the need for greater exertional stress protocols and rigorous experimental control of potential confounding factors when studying EIGS. In addition, the findings provide support for the need to assess a cluster of EIGS biomarkers commonly used in exercise gastroenterology research, due to the ease of application and low-medium invasiveness, that assess the impact of exercise on gastrointestinal status and systemic responses linked to performance and clinical implications (Costa et al., 2022).

However, it is important to note that due to participant availability within the respective original studies, the washout period between test and re-test was not consistent between participants, varying between 5- to 14-days. This may constitute a limitation in reliability of the current meta-data, warranting caution when interpreting reliability of single EIGS biomarker. Nevertheless, while this study provides necessary insights into reliability of markers when using exertional-stress models that modestly perturb gastrointestinal and immune responses, further exploration is warranted when using exercise protocols known to provoke clinically relevant changes to all biomarkers of EIGS (≥2 h in heat or ≥3 h duration).

Biomarkers of gastrointestinal epithelial integrity (e.g., I-FABP), lumen originated bacterial endotoxin translocation (e.g., sCD14 and LBP), and systemic immune responses (e.g., leukocyte counts, *in-vitro* neutrophil function, and systemic inflammatory cytokine profile) have widespread application in exercise-gastroenterology research and are critical in establishing the magnitude of EIGS and its degree of clinical significance. The current study provided a comprehensive reliability analysis, using paired *t*-tests (or non-parametric equivalents), Cohen’s *d* effect size, two-tailed correlation, and intra-class correlation coefficients, on a multitude of biomarkers in application of adequate exertional-stress (i.e., 2 h HIIT). All resting pre-exercise biomarkers presented with small effect sizes (*d* ≤ 0.40) and no differences between trials (*p* > 0.05). The most robust measures based on test-retest analysis were plasma concentrations of cortisol, LBP, and anti-inflammatory cytokines IL-10 and IL-1ra, which all exhibited good-to-excellent reliability (ICC) and strong correlations (*r* = 0.665). Plasma LBP concentration provides evidence of internal exposure to gram-negative bacterial endotoxin lipopolysaccharide, functioning as an indirect marker of luminal to systemic endotoxemia as a result of increased intestinal permeability (Costa et a., 2017b; Seethaler et al., 2021). Interestingly, a minimal effect size (*d* = 0.08), strong correlation (r = 0.813) and good ICC (ICC = 0.76) was associated with resting measures of plasma LBP concentration providing strong justification for its application as a primary biomarker of intestinal permeability and exercise associated endotoxemia. This notion is supported by a recent study that reported a strong correlation between resting plasma LBP concentration and the dual sugars lactulose/mannitol ratio, independent of age, BMI, and biological sex (Seethaler et al., 2021). The application of anti-inflammatory cytokines, IL-10 and IL-1ra, as principle biomarkers for measuring the degree of exercise-induced systemic inflammatory response is also warranted, because of: 1) reliability outcomes, 2) consistent response sensitivity in comparison to other systemic inflammatory profile cytokines, 3) consistency in response to exertional and exertional-heat stress models, and 4) magnitude of response links to clinical outcomes (i.e., pro- to anti-inflammatory ratio) (Costa et al., 2022). Both cytokines presented large correlations and good-to-excellent ICCs (ICC = 0.73 and ICC = 0.92, respectively). Elevations in these biomarkers indicate a transient systemic pro-inflammatory response (i.e., increased IL-1β and TNF-α) has occurred, characteristic of pathogenic luminal translocation into systemic circulation, resulting in activation of systemic inflammatory and compensatory anti-inflammatory responses (Gill et al., 2015a; Gill et al., 2015b; Peake et al., 2015; Gill et al., 2016b; Costa et al., 2022). Based on combined ICC and correlation analysis, alternative variables of EIGS (i.e., *in-vitro* neutrophil function, neutrophil counts, and plasma concentrations I-FABP, sCD14, and pro-inflammatory cytokines IL-1β and TNF-α) displayed moderate test-retest reliability, and despite minimal effect sizes, it is recommended that these biomarkers are used in combination and/or in conjunction with more reliable measures. Leukocyte counts exhibited the poorest reliability with no significant correlation (*r* = 0.327, *p* > 0.05) and a poor ICC (0.33); therefore, continued use is only justified as a supportive biomarker of established immune measures with caution required in interpretation and reporting.

A novel and benchmark outcome of this study is the establishment of MDC of each resting biomarker (Table 4), stipulating reference cut-off values that are indicative of true physiological change. In context of the strenuous exercise applied in this study, despite significant changes in several biomarkers pre-to post-exercise, no marker increased beyond the associated MDC. This confirms 2 h of HIIT is not as potent a stimulus of EIGS, as seen with longer exercise duration and/or heat exposure during exercise (Snipe et al., 2017; Snipe and Costa, 2018a; Snipe and Costa, 2018b; Gaskell et al., 2021c; Gaskell et al., 2021d), and confirms the concerns highlighted in a recent methodological review by Costa et al. (2022). Of particular interest is the MDC associated with I-FABP, a measure applied consistently as a principle marker of gastrointestinal perturbations within exercise gastroenterology research. Previously a change of *≥*1000 pg/ml from baseline was understood to reflect significant acute gastrointestinal integrity disturbances of clinical significance (Pelsers et al., 2005; Haas et al., 2009; Jekarl et al., 2015; Surbatovic et al., 2015; Al-Saffar et al., 2017; Linsalata et al., 2018; Martinez-Fierro et al., 2019; Power et al., 2021), seen consistently as a result of aggressive exercise protocols (2 h exertional heat-stress or ≥3 h duration) (Snipe and Costa, 2018a; Snipe and Costa, 2018b; Snipe & Costa, 2018b; Gaskell et al., 2019; Gaskell et al., 2021a). However, findings from this study reveal that in effect, a change of ≥1300 pg/ml, is necessary to confirm true physiological change as opposed to a change attributable to measurement error. Amidst the establishment of test-retest reliability of EIGS variables, MDC reference values function to support accurate translation of research findings into practice and safeguard against prospective misinterpretation and overstated conclusions that have been a component of exercise-gastroenterology research in the past (Snipe et al., 2018b).

In contrast with resting measures, test-retest reliability of exercise-associated magnitude response was highly variable, and while most magnitude response measures demonstrated no difference between trials (aside from plasma IL-10 concentration), and small effect sizes, correlation and ICC analysis revealed biomarkers to have either poor-to-moderate reliability (i.e., plasma cortisol and I-FABP concentration, leukocyte and neutrophil counts) or no reliability (plasma sCD14, LBP, IL-1β, TNF-α, and IL-1ra concentrations). *In-vitro* bacterially-stimulated elastase was the only marker that displayed good test-retest reliability (ICC = 0.72), with moderate-good correlation (*r* = 0.646). Although, a lower mean HR and RPE was reported in Trial-2 *vs*. Trial-1, likely due to a trial order effect, it did not appear to influence the overall physiological and thermal strain, which are the key aspects of physiological stress likely to impact the magnitude of EIGS biomarkers (Costa et al., 2017b; Costa R. J. S. et al., 2020; Costa et al., 2022). In view of the relatively modest impact of 2 h HIIT on gastrointestinal disturbances, poorer test-retest reliability is expected to be observed when applying more aggressive exercise-stress models (i.e., ≥2 h in heat), attributable to commonly observed increases in intra- and inter-variation between individuals. In addition, despite rigorous control of the experimental protocol in the current study, as per Costa et al. (2022), controlling for this variability evidently remains challenging. Accordingly, the SEM and MDC associated with magnitude of change response biomarkers were deemed irrelevant and not included in analysis, with use of MDC reference values related to resting measures recommended when verifying if a change from pre-to post-exercise is of clinical relevance.

Despite the widespread application of these biomarkers in exercise-gastroenterology research, only one other study has previously reported on reliability characteristics of EIGS biomarkers in relation to exercise-stress (Ogden et al., 2020a). Unfortunately, some limitations were associated with the study design, including not applying an exercise protocol sufficient to cause any substantial EIGS biomarker response (80 min walking protocol). This likely explains the small magnitude response in assessed biomarkers and inevitability of less response variability, and subsequent suggestion of adequate reliability of assessed markers. The experimental limitation deems post-exercise and magnitude response reliability analysis translationally limited, making it challenging to appraise in the context of the current findings. Furthermore, the reliability analysis was performed on a limited number of biomarkers (i.e., I-FABP, LBP, dual-sugar test, claudin-3, total 16S DNA concentrations and *Bacteriodes*/total bacterial DNA), with no systemic cytokines indicative of luminal pathogenic translocation of clinical relevance included, and some included markers recognised to provide erroneous outcomes and prone to misinterpretation (Costa et al., 2022). For example, *Bacteriodes*/total bacterial DNA, is a non-established measure that was used to quantify whole bacteria translocation from lumen to systemic circulation, and was reported to have poor-reliability (Ogden et al., 2020b). The current study expanded on the concept of whole bacteria lumen to systemic circulation translocation by measuring and reporting full plasma bacterial composition profile in addition to total bacterial DNA concentration profile in response to more substantial exercise stress. The findings support no Trial-1 *vs*. Trial-2 differences in pre-exercise resting and post-exercise bacterial DNA concentration with no-to-low Cohen’s *d* effect size, but no significant two-tail correlation and poor ICC outcomes. In addition, plasma bacteria α-diversity at phyla, family, and genus level presented similar outcomes for comparative tests and effect size, with no resting and exercise-induced magnitude response two-tail correlation and poor ICC outcomes. Surprisingly, regardless of methodological discrepancies, both studies displayed robust reliability for pre-exercise resting LBP measures (*d* = .07, *r* = 0.85 *vs*. *d* = .08, *r* = 0.813), but this did not translate to exercise-associated Δ magnitude response (*d* = .39, *r* = −0.16 *vs*. *d* = .11, *r* = 0.119). In contrast to findings from the current study, plasma I-FABP displayed strong reliability at rest (*r* = 0.750) (Ogden et al., 2020a), compared to weak test-retest correlations reported at the same time point in the current study (*r* = 0.411). ICC analysis further substantiated a weak test-retest reliability with an ICC of 0.21 pre-exercise. Contrasting findings between the two studies are not unexpected considering the differences in exercise protocols and control measures. It is expected that test-retest reliability of these biomarkers in response to exercise will further lessen when applying more potent exertional stress models attributable to the commonly observed intra- and inter-variation in gastrointestinal perturbations (i.e., the greater the exercise associated gastrointestinal perturbation, the higher the likelihood of individual variability) (Costa et al., 2022). To confirm or contrast this assumption, future reliability analysis is required in well-controlled studies using exercise models that mimick real-world exercise activities that consistently report incidence and greater severity of gastrointestinal issues (e.g., ≥2 h heat or ≥3 h, and/or ultra-endurance based activities).

Metabolic by-products of commensal bacteria (e.g., SCFA-acetate, butyrate and propionate) along the gastrointestinal tract are known to maintain intestinal epithelial integrity through regulation of tight junctions, enhancement of epithelial cell stability, and reduction in permeability In turn, there is a reduced risk of translocation of pathogenic agents within the intestinal lumen into circulation, attenuating local and systemic inflammatory responses (Sekirov et al., 2010; Canani et al., 2011; Gilert et al., 2018). In the current study, it was observed that increased concentrations of plasma butyrate pre-exercise, was associated with reduced exercise-associated perturbations to the intestinal epithelium, as evidenced by lower concentrations of plasma I-FABP post-exercise. In addition, higher fecal acetate concentrations pre-exercise was linked to higher plasma concentrations of pro-inflammatory cytokines, IL-1β and TNF-α; however, the total magnitude of response of IL-1β and TNF-α to the exercise protocol was insignificant and of no clinical relevance. Other plasma and fecal SCFA concentrations showed little to no association with EIGS biomarkers following 2 h HIIT. It is the authors understanding that the current study is the first to directly assess the association between SCFAs and markers of EIGS. While it is acknowledged that correlations do not infer an effect, it is not unexpected that butyrate may offer some protection against intestinal epithelial injury considering its positive physiological effects at intestinal level that have been observed consistently *in-vitro* and *in-vivo*, in both general and clinical populations (Mariadason et al., 1997; Canani et al., 2011; Clarke et al., 2011). More recent studies using human-exercise models have hypothesised the potential of SCFAs to attenuate EIGS. Bennett et al. (2020) observed that greater *a*-diversity and relative SCFA-producing commensal bacteria attenuates the degree of EIGS experienced by endurance-trained athletes exercising for 2 h in hot conditions. While not directly investigating SCFAs impact on gut barrier integrity, the authors theorised the increased SCFA production associated with higher relative abundance of commensal bacteria was responsible for reduced EIGS. Interpretation of findings from the current study should be done in the context of variable test-retest reliability of SCFA biomarkers ranging from poor-to-moderate for both fecal and plasma measures. The relatively low reliability may be indicative of the sensitivity of these measures to confounding factors and large inter- and intra-subject variability. Further exploration is warranted in order to identify these potential confounders of plasma and fecal SCFA levels, with special consideration to plasma butyrate given findings from the current study and the understanding of the role butyrate may have in maintaining epithelial cell integrity. It is suggested that future studies start by adopting an extended pre-trial dietary control protocol to help reduce some variability in these measures.

Bacterial endotoxin translocation is well-understood to be a key instigator of transient systemic inflammation during and following substantial exertional load (Costa et al., 2017b; Costa R. J. S. et al., 2020), but whole bacteria luminal to systemic translocation is a novel concept that warranted further exploration with scarce research to date and nothing comprehensive in the exercise research setting (Castillo et al., 2019; Costa et al., 2022; Villarroel et al., 2022). The current study is the first to comprehensively explore exercise-associated bacteremia (i.e., concentration and composition) using an exertional stress model reflective of real-life practices of endurance and team-sports athletes that have the potential to foster gastrointestinal symptoms (Steege et al., 2008; Engebretsen et al., 2013). Findings demonstrate that 2 h of HIIT results in a significant increase in plasma bacterial DNA concentration from pre-to post-exercise. Interpretation of bacterial presence in circulation warrants some caution however, due to the contamination risk associated with sample handling (i.e., sample collection, processing and analysis procedures), posing a challenge in distinguishing between true biological bacterial DNA in plasma and that related to contaminated consumables and samples (Nikkari et al., 2001). To minimise the risk of cross-contamination and including artefact values in data analysis, risk management procedures were employed as described in the methods section and only bacterial groups with a conservative ≥0.5% relative abundance, respective to the determination medium, were included for data analysis as per methods and proposed in previous studies (Bokulich et al., 2013; Lou et al., 2021). Plus, the included blank control samples (i.e., pyrogen and DNAse/RNAse from water) underwent the same processing procedures, yielding negative detectable outcomes. Furthermore, resting levels of plasma bacterial DNA have significantly low biomass compared to that residing in the gastrointestinal tract, presenting a risk of DNA falling below the detectable limit. In the current study, while the total plasma DNA concentration of some participants pre-exercise was undetected (i.e., below the minimal detection level of <0.02 ng/μl), plasma DNA concentration was quantifiable for all participants post-exercise consistent across trials validating a true increase in total plasma DNA, likely with substantial attributions from whole bacteria lumen to circulating translocation. Lastly, the authors acknowledge that exercise-induced cellular damage and consequential increases in cellular free DNA can lead to misinterpretation of findings (Agassi et al., 2015), which have been overcome in the current study through determination of bacterial composition profile. Findings demonstrated significant changes to relative abundance of certain phyla, family and genus groups from pre-to post-exercise protocol, consistent across trials, providing further evidence of bacterial translocation. Preceding studies, albeit scarce, showed dissimilar pre- to post-exercise plasma bacterial profile outcomes, likely related to differing exertional stress models and measurement methodologies and data presentation (e.g., total bacterial s16 DNA to *Bacteroides* ratio) (March et al., 2019; Ogden et al., 2020a, 2020b). A novel finding in the current study was that *Proteobacteria* (phyla, family and genus), and not *Bacteroides* and *Firmicutes,* demonstrated the greatest translocation, creating significant shifts in alpha diversity (i.e., reduction in alpha diversity at phyla level associated with increased *Proteobacteria*). As a result, a focus on *Proteobacteria* as a measure of bacterial translocation is justified over *Bacteriodes*. In support of previous research of the healthy blood microbiome, *Proteobacteria* was the most dominant phyla, accounting for 63% of the bacterial composition pre-exercise (Castillo et al., 2019). Mechanistically, *Proteobacteria* and the healthy *vs*. disease state blood microbiome is still poorly understood. Previous studies, however, have consistently highlighted a correlation between gut dysbiosis (i.e., inflammation associated with IBD) and increased *Proteobacteria* in the gastrointestinal tract, with the notion it may play a pro-inflammatory role in disease states (Frank et al., 2007; Mukhopadhya et al., 2012; Matsuoka and Kanai, 2014; Rizzatti et al., 2017; Caruso et al., 2020). It is hypothesised that EIGS-associated transient inflammation of the gastrointestinal tract stimulates an increase in luminal *Proteobacteria* concentration and subsequent translocation into plasma, offering a potential explanation for the high relative abundance of *Proteobacteria* phyla, family and genus in circulation. Of interest would be post-exercise endoscopic findings that reflect acute transient changes occurring at the terminal ilium (i.e., where epithelial cell lining is thin and subject to damage), which would perhaps demonstrate increased *Proteobacteria* concentrations related to exercise-induced gut disturbances. Unfortunately, post-exercise fecal samples are limited due to bacterial DNA dominating the sigmoid colon, and not the critical point at the terminal ilium. To confirm and expand on findings from the current study, further research using sufficient exercise stress models reflective of real-life training and competition of endurance and/or ultra-endurance athletes that suffer from gastrointestinal issues during exercise is needed, with exploration in the implications of changes in bacteremia on Ex-GIS, performance and clinical outcomes.

## Conclusion

The 2 h HIIT protocol was sufficient in inducing perturbations to certain biomarkers of EIGS, consistent across both trials. EIGS-associated disturbances also initiated luminal to systemic translocation of whole bacteria, evidenced by an increase in total DNA concentrations pre-to post-exercise and alterations in the blood microbiome profile. The potential impact of significant bacterial translocation during endurance exercise on Ex-GIS, performance and health still needs to be explored. Extensive test-retest analysis exposed that commonly used biomarkers of EIGS have variable reliability, ranging from poor-to-excellent. It is, therefore, strongly advised that a cluster of biomarkers is used and interpreted collectively when assessing EIGS, and/or general gastrointestinal integrity and systemic responses to exercise. The application of a limited number of measures risks erroneous interpretations due to large variability within and between individuals in response to exercise. A limitation to this study was the use of an exercise protocol that only moderately perturbed select EIGS biomarkers, making it challenging to accurately evaluate the reliability of markers when measuring magnitude of response. Of value would be repeating similar reliability analysis using more substantial exertional-stress models well-established in inducing significant changes to EIGS biomarkers, using the current minimal detectable change (MDC) as guidance. Lastly, a moderate-negative correlation was observed between pre-exercise plasma butyrate and concentrations of plasma I-FABP post-exercise, suggesting an association between higher levels of butyrate and reduced exercise-associated perturbations to the intestinal epithelium. Further research is warranted to confirm this relationship and investigate if SCFA concentrations have a role in attenuating the severity of EIGS.

## Data Availability

The datasets presented in this study can be found in online repositories. The names of the repository/repositories and accession number(s) can be found below: https://www.ncbi.nlm.nih.gov/sra/PRJNA926792.

## References

[B1] Agassi R. Czeiger D. Shaked G. Avriel A. Sheynin J. Lavrenjov K. (2015). Measurement of circulating cell-free DNA levels by a simple fluorescent test in patients with breast cancer. Am. J. Clin. Pathol. 143, 18–24. 10.1309/AJCPI5YHG0OGFAHM 25511138

[B2] Al-Saffar A. K. Meijer C. H. Gannavarapu V. R. Hall G. Li Y. Tartera D. (2017). Parallelchanges in Harvey-Bradshaw index, TNF*α*, and intestinal fatty acid binding protein in response to infliximab in crohn's disease. Gastroenterol. Res. Pract. 2017, 1745918. 10.1155/2017/1745918 29201046 PMC5672611

[B4] Bennett C. J. Henry R. Snipe R. M. J. Costa R. J. S. (2020). Is the gut microbiota bacterial abundance and composition associated with intestinal epithelial injury, systemic inflammatory profile, and gastrointestinal symptoms in response to exertional-heat stress? J. Sci. Med. Sport 23, 1141–1153. 10.1016/j.jsams.2020.06.002 32620352

[B5] Bokulich N. A. Subramanian S. Faith J. J. Gevers D. Gordon J. I. Knight R. (2013). Quality-filtering vastly improves diversity estimates from Illumina amplicon sequencing. Nat. Methods 10, 57–59. 10.1038/nmeth.2276 23202435 PMC3531572

[B6] Canani R. B. Costanzo M. D. Leone L. Pedata M. Meli R. Calignano A. (2011). Potential beneficial effects of butyrate in intestinal and extraintestinal diseases. World J. Gastroenterol. 17, 1519–1528. 10.3748/wjg.v17.i12.1519 21472114 PMC3070119

[B7] Caruso R. Lo B. C. Nunez G. (2020). Host-microbiota interactions in inflammatory bowel disease. Nat. Rev. Immunol. 20, 411–426. 10.1038/s41577-019-0268-7 32005980

[B8] Castillo D. J. Rifkin R. F. Cowan D. A. Potgieter M. (2019). The healthy human blood microbiome: Fact or fiction*?* Front. Cell. Infect. Microbiol. 9, 148. 10.3389/fcimb.2019.00148 31139578 PMC6519389

[B9] Clarke J. M. Topping D. L. Christophersen C. T. Bird A. R. Lange K. Saunders I. (2011). Butyrate esterified to starch is released in the human gastrointestinal tract. Am. J. Clin. Nutr. 94, 1276–1283. 10.3945/ajcn.111.017228 21940597

[B10] Clauss M. Gérard P. Mosca A. Leclerc M. (2021). Interplay between exercise and gut microbiome in the context of human health and performance. Front. Nutr. 8 8. 10.3389/fnut.2021.637010 PMC8222532 34179053

[B11] Costa R. J. S. Camões-Costa V. Snipe R. M. J. Dixon D. Russo I. Huschtscha Z. (2019). Impact of exercise-induced hypohydration on gastrointestinal integrity, function, symptoms, and systemic endotoxin and inflammatory profile. J. Appl. Physiol. 126, 1281–1291. 10.1152/japplphysiol.01032.2018 30896356

[B12] Costa R. J. S. Camões-Costa V. Snipe R. M. J. Dixon D. Russo I. Huschtscha Z. (2020b). The impact of a dairy milk recovery beverage on bacterially-stimulated neutrophil function and gastrointestinal tolerance in response to hypohydration inducing exercise stress. Int. J. Sport Nutr. Exerc. Metab. 30 (4), 237–248. 10.1123/ijsnem.2019-0349 32460239

[B13] Costa R. J. S. Crockford M. J. Moore J. P. Walsh N. P. (2014). Heat acclimation responses of an ultra-endurance running group preparing for hot desert-based competition. Eur. J. Sport Sci. 14, S131–S141. 10.1080/17461391.2012.660506 24444197

[B14] Costa R. J. S. Gaskell S. K. McCubbin A. J. Snipe R. M. J. (2020a). Exertional-heat stress-associated gastrointestinal perturbations during olympic sports: Management strategies for athletes preparing and competing in the 2020 tokyo olympic games. Temperature 7, 58–88. 10.1080/23328940.2019.1597676 PMC7053925 32166105

[B15] Costa R. J. S. Miall A. Khoo A. Rauch C. Snipe R. M. J. Camões-Costa V. (2017a). Gut-training: The impact of two weeks repetitive gut-challenge during exercise on gastrointestinal status, glucose availability, fuel kinetics, and running performance. Appl. Physiol. Nutr. Metab. 42, 547–557. 10.1139/apnm-2016-0453 28177715

[B16] Costa R. J. S. Oliver S. J. Laing S. J. Walters R. Bilzon J. L. Walsh N. P. (2009). Influence of timing of postexercise carbohydrate-protein ingestion on selected immune indices. Int. J. Sport Nutr. Exerc. Metab. 19, 366–384. 10.1123/ijsnem.19.4.366 19827462

[B17] Costa R. J. S. Snipe R. Camões-Costa V. Scheer B. V. Murray A. (2016). The impact of gastrointestinal symptoms and dermatological injuries on nutritional intake and hydration status during ultramarathons. Sports Med. Open 2, 1–14. 10.1186/s40798-015-0041-9 PMC4701764 26767151

[B18] Costa R. J. S. Snipe R. M. J. Kitic C. M. Gibson P. R. (2017b). Systematic review: Exercise-induced gastrointestinal syndrome – implications for health and intestinal disease. Aliment. Pharmacol. Ther. 46, 246–265. 10.1111/apt.14157 28589631

[B19] Costa R. J. S. Walters R. Bilzon J. L. Walsh N. P. (2011). Effects of immediate post exercise carbohydrate ingestion with and without protein on neutrophil degranulation. Int. J. Sport Nutr. Exerc. Metab. 21, 205–213. 10.1123/ijsnem.21.3.205 21719901

[B20] Costa R. J. S. Young P. Gill S. K. Snipe R. M. J. Gaskell S. Russo I. (2022). Assessment of exercise-associated gastrointestinal perturbations in research and practical settings: Methodological concerns and recommendations for best practice. Int. J. Sport Nutr. Exerc. Metab. 13, 387–418. 10.1123/ijsnem.2022-0048 35963615

[B21] Dill D. B. Costill D. L. (1974). Calculation of percentage changes in volumes of blood, plasma, and red cells in dehydration. J. Appl. Physiol. 37, 247–248. 10.1152/jappl.1974.37.2.247 4850854

[B22] Engebretsen L. Soligard T. Steffen K. Alonso J. M. Aubry M. Budgett R. (2013). Sports injuries and illnesses during the london summer olympic games 2012. Br. J. Sports Med. 47, 407–414. 10.1136/bjsports-2013-092380 23515712

[B23] Frank D. N. Amand St Feldman R. A. Boedeker E. C. Harpaz N. Pace N. R. (2007). Molecular-phylogenetic characterization of microbial community imbalances in human inflammatory bowel diseases. Proc. Natl. Acad. Sci. U. S. A. 104, 13780–13785. 10.1073/pnas.0706625104 17699621 PMC1959459

[B24] Gaskell S. K. Gill P. Muir J. Henry R. Costa R. J. S. (2021d). Impact of 24h low and high FODMAP diet on faecal and plasma short chain fatty acid concentration, and its influence on markers of exercise-induced gastrointestinal syndrome in response to exertional-heat stress. Nutr. Diet. 78, 8.

[B25] Gaskell S. K. Rauch C. Costa R. J. S. (2021a). Gastrointestinal assessment and management procedures for exercise-associated gastrointestinal symptoms. Aspetar Sports Med. J. 10, 36–44.

[B26] Gaskell S. K. Rauch C. Costa R. J. S. (2021b). Gastrointestinal assessment and therapeutic intervention for the management of exercise-associated gastrointestinal symptoms: A case series translational and professional practice approach. Front. Physiol. 12, 719142. 10.3389/fphys.2021.719142 34557109 PMC8452991

[B27] Gaskell S. K. Rauch C. Parr A. Costa R. J. (2021c). Diurnal versus nocturnal exercise – effect on the gastrointestinal tract. Med. Sci. Sports Exerc. 53, 1056–1067. 10.1249/MSS.0000000000002546 33065594

[B28] Gaskell S. K. Snipe R. M. Costa R. J. (2019). Test-retest reliability of a modified visual analog scale assessment tool for determining incidence and severity of gastrointestinal symptoms in response to exercise stress. Int. J. Sport Nutr. Exerc. Metab. 29, 411–419. 10.1123/ijsnem.2018-0215 30632417

[B29] Gilbert J. A. Blaser M. J. Caporaso J. G. Jansson J. K. Lynch S. V. Knight R. (2018). Current understanding of the human microbiome. Nat. Med. 24, 392–400. 10.1038/nm.4517 29634682 PMC7043356

[B30] Gill P. A. van Zelm M. C. Ffrench R. A. Muir J. G. Gibson P. R. (2020). Successful elevation of circulating acetate and propionate by dietary modulation does not alter T-regulatory cell or cytokine profiles in healthy humans: A pilot study. Eur. J. Nutr. 59, 2651–2661. 10.1007/s00394-019-02113-2 31650328

[B31] Gill S. K. Allerton D. M. Ansley-Robson P. Hemming K. Cox M. Costa R. J. S. (2016a). Does acute high dose probiotic supplementation containing lactobacillus casei attenuate exertional-heat stress induced endotoxaemia and cytokinaemia?. Int. J. Sport Nutr. Exerc. Metab. 26, 268–275. 10.1123/ijsnem.2015-0186 26568577

[B32] Gill S. K. Hankey J. Wright A. Marczak S. Hemming K. Allerton D. M. (2015b). The impact of a 24-hour ultra-marathon on circulatory endotoxin and cytokine profile. Int. J. Sports *Med.* 36, 688–695. 10.1055/s-0034-1398535 25941924

[B33] Gill S. K. Price M. Costa R. J. (2016b). Measurement of saliva flow rate in healthy young humans: Influence of collection time and mouthrinse water temperature. Eur. J. Oral Sci. 124, 447–453. 10.1111/eos.12294 27671982

[B34] Gill S. K. Teixeira A. Rama L. Rosado F. Hankey J. Scheer V. (2015a). Circulatory endotoxin concentration and cytokine profile in response to exertional-heat stress during a multi-stage ultra-marathon competition. Exerc. Immunol. Rev. 21, 114–128. 25830597

[B35] Haas V. Büning C. Buhner S. von Heymann C. Valentini L. Lochs H. (2009). Clinical relevance of measuring colonic permeability. Eur. J. Clin. Invest. 39, 139–144. 10.1111/j.1365-2362.2008.02075.x 19200167

[B37] Imdad S. Lim W. Kim J. Kang C. (2022). Intertwined relationship of mitochondrial metabolism, gut microbiome and exercise potential. Int. J. Mol. Sci. 28 23, 2679. 10.3390/ijms23052679 PMC8910986 35269818

[B38] Jekarl D. W. Kim J. Y. Lee S. Kim M. Kim Y. Han K. (2015). Diagnosis and evaluation of severity of sepsis via the use of biomarkers and profiles of 13 cytokines: A multiplex analysis. Clin. Chem. Lab. Med. 53, 575–581. 10.1515/cclm-2014-0607 25274957

[B39] Koo T. K. Li M. Y. (2016). A guideline of selecting and reporting intraclass correlation coefficients for reliability research. J. Chiropr. Med. 15, 155–163. 10.1016/j.jcm.2016.02.012 27330520 PMC4913118

[B40] Linsalata M. Riezzo G. D’Attoma B. Clemente C. Orlando A. Russo F. (2018). Noninvasive biomarkers of gut barrier function identify two subtypes of patients suffering from diarrhoea predominant-IBS: A case-control study. BMC Gastroenterol. 18, 167. 10.1186/s12876-018-0888-6 30400824 PMC6219148

[B41] Luo Z. Alekseyenko A. V. Ogunrinde E. Li M. Li Q-Z. Huang L. (2021). Rigorous plasma microbiome analysis method enables disease association discovery in clinic. Front. Microbiol. 11 11, 613268. 10.3389/fmicb.2020.613268 PMC7820181 33488555

[B42] March D. S. Jones A. W. Thatcher R. Davison G. (2019). The effect of bovine colostrum supplementation on intestinal injury and circulating intestinal bacterial DNA following exercise in the heat. Eur. J. Nutr. 58, 1441–1451. 10.1007/s00394-018-1670-9 29574607 PMC6561991

[B43] Mariadason J. M. Barkla D. H. Gibson P. R. (1997). Effect of short-chain fatty acids on paracellular permeability in Caco-2 intestinal epithelium model. Am. J. Physiol. 272, 705–712. 10.1152/ajpgi.1997.272.4.G705 9142899

[B44] Martinez-Fierro M. L. Garza-Veloz I. Rocha-Pizaña M. R. Cardenas-Vargas E. Cid-Baez M. A. Trejo-Vazquez F. (2019). Serum cytokine, chemokine, and growth factor profiles and their modulation in inflammatory bowel disease. Medicine 98, e17208. 10.1097/MD.0000000000017208 31567972 PMC6756690

[B45] Matsuoka K. Kanai T. (2014). The gut microbiota and inflammatory bowel disease. Semin. Immunopath. 37, 47–55. 10.1007/s00281-014-0454-4 PMC4281375 25420450

[B47] Mukhopadhva I. Hansen R. El-Omar E. M. Hold G. L. (2012). IBD-what role do *Proteobacteria* play? Nat. Rev. Gastroenterol. Hepatol. 219, 219–230. 10.1038/nrgastro.2012.14 22349170

[B48] Nabizadeh E. Sadeghi J. Ahangarzadeh Rezaee M. Hasani A. Samadi Kafil H. Ghotaslou A. (2022). Interaction between altered gut microbiota and sepsis: A hypothesis or an authentic fact. J. Intensive Care Med. 38, 121–131. 10.1177/08850666221102796 35603752

[B49] Nikkari S. McLaughlin I. J. Bi W. Dodge D. E. Relman D. A. (2001). Does blood of healthy subjects contain bacterial ribosomal DNA? J. Clin. Microbiol. 39, 1956–1959. 10.1128/JCM.39.5.1956-1959.2001 11326021 PMC88056

[B50] Ogden H. B. Fallowfield J. L. Child R. B. Davison G. Fleming S. C. Delves S. K. (2022). Acute _L_-glutamine supplementation does not improve gastrointestinal permeability, injury or microbial translocation in response to exhaustive high intensity exertional-heat stress. Eur. J. Sport Sci. 22, 1865–1876. 10.1080/17461391.2021.2001575 34726114

[B51] Ogden H. B. Fallowfield J. L. Child R. B. Davison G. Fleming S. C. Delves S. K. (2020b). Influence of aerobic fitness on gastrointestinal barrier integrity and microbial translocation following a fixed-intensity military exertional heat stress test. Eur. J. Appl. Physiol. 120, 2325–2337. 10.1007/s00421-020-04455-w 32794058

[B52] Ogden H. B. Fallowfield J. L. Child R. B. Davison G. Fleming S. C. Edinburgh R. M. (2020a). Reliability of gastrointestinal barrier integrity and microbial translocation biomarkers at rest and following exertional heat stress. Physiol. Rep. 8, 143744–e15174. 10.14814/phy2.14374 PMC7070100 32170836

[B54] Peake J. M. Gatta P. D. Suzuki K. Nieman D. C. (2015). Cytokine expression and secretion by skeletal muscle cells: Regulatory mechanisms and exercise effects. Exer. Immunol. Rev. 21, 8–25. 25826432

[B55] Pelsers M. M. Hermens W. T. Glatz J. F. (2005). Fatty acid-binding proteins as plasma markers of tissue injury. Clin. Chim. Acta. 352, 15–35. 10.1016/j.cccn.2004.09.001 15653098

[B56] Power N. Turpin W. Espin-Garcia O. Croitoru K. CCC GEM Project Research Consortium (2021). CCC GEM Project Research Consortium, & Croitoru, KSerum zonulin measured by commercial kit fails to correlate with physiologic measures of altered gut permeability in first degree relatives of Crohn's disease patients. Front. Physiol. 12, 645303. 10.3389/fphys.2021.645303 33841181 PMC8027468

[B57] Quast C. Pruesse E. Yilmaz P. Gerken J. Schweer T. Yarza P. (2013). & glӧckner, F. OThe SILVA ribosomal RNA gene database project: Improved data processing and web-based tools. Nucleic Acids Res. 41, 590–596. 10.1093/nar/gks1219 PMC3531112 23193283

[B58] Rizzatti G. Lopetuso L. R. Gibiino G. Binda C. Gasbarrini A. (2017). Proteobacteria: A common factor in human diseases. Biomed. Res. Int. 9 2017, 9351507. 10.1155/2017/9351507 PMC5688358 29230419

[B59] Russo I. Camoes-Costa V. Gaskell S. K. Porter J. Burke L. M. Costa R. J. S. (2019). Systematic literature review: The effect of diary milk on markers of recovery optimisation in response to endurance exercise. Int. J. Sports *Sci. Coach* 9, 69–85. 10.5923/j.sports.20190904.01

[B60] Russo I. Della Gatta P. A. Garnham A. Porter J. Burke L. M. Costa R. J. S. (2021a). Assessing overall exercise recovery processes using carbohydrate and carbohydrate-protein containing recovery beverages. Front. Physiol. 12, 50. 10.3389/fphys.2021.628863 PMC7890126 33613323

[B61] Russo I. Della Gatta P. A. Garnham A. Porter J. Burke L. M. Costa R. J. S. (2021b). Does the nutritional composition of dairy milk based recovery beverages influence post-exercise gastrointestinal and immune status, and subsequent markers of recovery optimisation in response to high intensity interval exercise? Front. Nutr. 7, 343. 10.3389/fnut.2020.622270 PMC7840831 33521041

[B62] Russo I. Della Gatta P. A. Garnham A. Porter J. Burke L. M. Costa R. J. S. (2021c). The effects of an acute "Train-Low" nutritional protocol on markers of recovery optimization in endurance-trained male athletes. Int. J. Sports Physiol. Perform. 16, 1764–1776. 10.1123/ijspp.2020-0847 34044369

[B63] Seethaler B. Basrai M. Neyrinck A. M. Nazare J-A. Walter J. Delzenne N. M. (2021). Biomarkers for assessment of intestinal permeability in clinical practice. Am. J. Physiol. Gastrointest. Liver Physiol. 321, 11–17. 10.1152/ajpgi.00113.2021 34009040

[B64] Sekirov I. Russell S. L. Antunes L. C. M. Finlay B. B. (2010). Gut microbiota in health and disease. Physiol. Rev. 90, 859–904. 10.1152/physrev.00045.2009 20664075

[B65] Snipe R. M. J. Costa R. J. S. (2018a). Does biological sex impact intestinal epithelial injury, small intestine permeability, gastrointestinal symptoms and systemic cytokine profile in response to exertional-heat stress? J. Sports Sci. 36, 2827–2835. 10.1080/02640414.2018.1478612 29790452

[B66] Snipe R. M. J. Costa R. J. S. (2018b). Does the temperature of water ingested during exertional-heat stress influence gastrointestinal injury, symptoms, and systemic inflammatory profile? J. Sci. Med. Sport 21, 771–776. 10.1016/j.jsams.2017.12.014 29371075

[B67] Snipe R. M. J. Khoo A. Kitic C. M. Gibson P. R. Costa R. J. S. (2017). Carbohydrate and protein intake during exertional heat stress ameliorates intestinal epithelial injury and small intestine permeability. Appl. Physiol. Nutr.Metab. 42, 1283–1292. 10.1139/apnm-2017-0361 28777927

[B68] Snipe R. M. J. Khoo A. Kitic C. M. Gibson P. R. Costa R. J. S. (2018b). The impact of exertional-heat stress on gastrointestinal integrity, gastrointestinal symptoms, systemic endotoxin and cytokine profile. Eur. J. Appl. Physiol. 118, 389–400. 10.1007/s00421-017-3781-z 29234915

[B69] Snipe R. M. J. Khoo A. Kitic C. M. Gibson P. R. Costa R. J. S. (2018a). The impact of mild heat stress during prolonged running on gastrointestinal integrity, gastrointestinal symptoms, systemic endotoxin and cytokine profiles. Int. J. Sports Med. 39, 255–263. 10.1055/s-0043-122742 29415294

[B70] Steege R. W. Van der Palen J. Kolkman J. J. (2008). Prevalence of gastrointestinal complaints in runners competing in a long-distance run: An internet-based observational study in 1281 subjects. Scand. J. Gastroenterol. 43, 1477–1482. 10.1080/00365520802321170 18777440

[B71] Surbatovic M. Popovic N. Vojvodic D. Milosevic I. Acimovic G. Stojicic M. (2015). Cytokine profile in severe Gram-positive and Gram-negative abdominal sepsis. Sci. Rep. 5, 11355. 10.1038/srep11355 26079127 PMC4468818

[B72] Villarroel J. Donkin I. Champion C. Burcelin R. Romain B. (2022). Endurance training in humans modulates the bacterial DNA signature of skeletal muscle. Biomedicines 10, 64. 10.3390/biomedicines10010064 PMC8773292 35052744

[B73] Walter E. Gibson O. R. Stacey M. Hill N. Parsons I. T. Woods D. (2021). Changes in gastrointestinal cell integrity after marathon running and exercise-associated collapse. Eur. J. Appl. Physiol. 121, 1179–1187. 10.1007/s00421-021-04603-w 33512586

[B74] Weir J. P. (2005). Quantifying test-retest reliability using the intraclass correlation coefficient and the SEM. J. Strength Cond. Res. 19, 231–240. 10.1519/15184.1 15705040

