# Supplementary material for: Reliability of pathophysiological markers reflective of exercise-induced gastrointestinal syndrome (EIGS) in response to 2-h high-intensity interval exercise: A comprehensive methodological efficacy exploration
Source: Front Physiol. 2023 Feb 21;14:1063335. doi: 10.3389/fphys.2023.1063335 (PMC9989174; doi:10.3389/fphys.2023.1063335)
Supplement: Supplementary file 1 [file DataSheet1.PDF]

Supplementary Table 1. Reliability indices of pre- to post-exercise magnitude of change in exercise-induced gastrointestinal syndrome (EIGS) biomarkers in response to 2 h high intensity interval exercise in temperate ambient conditions performed on two separate occasions.

	**Intraclass correlation coefficient *r*** [a]	**95% CI**	**p**
Cortisol (nMol/L)	0.41	0.09 to 0.66	**.008**
IFABP (pg/ml)	0.21	-0.16 to 0.52	.129
sCD14 (µg/ml)	-0.08	-0.42 to 0.28	.662
LBP (µg/ml)	0.19	-0.17 to 0.51	.149
Total leukocyte counts ($x10^9$/L)	0.48	0.18 to 0.70	**.002**
Neutrophil counts ($x10^9$/L)	0.46	0.16 to 0.69	**.002**
Total stimulated elastase (ng/ml)	0.72	0.49 to 0.86	**<.001**
Elastase release per cell (fg/ml)	0.23	-0.11 to 0.53	.097
IL-1β (pg/ml)	-0.15	-0.47 to 0.20	.797
TNF-α (pg/ml)	-0.26	-0.56 to 0.09	.933
IL-10 (pg/ml)	0.48	0.18 to 0.70	**.001**
IL-1ra (pg/ml)	0.16	-0.19 to 0.48	.183
Plasma bacterial DNA (ng/µl)	0.43	-0.09 to 0.76	.053
Plasma phyla SEI	0.46	0.006 to 0.77	**.020**
Plasma family SEI	0.46	-0.07 to 0.78	**.041**
Plasma genus SEI	0.18	-0.28 to 0.60	.236

[a] Intraclass correlation coefficient (ICC), whereby $r^a < 0.5$, $r^a = 0.5$-0.74, $r^a = 0.75$-0.9, and $r^a > 0.90$ for poor, moderate, good and excellent reliability respectively and 95% confidence intervals (CI).

Supplementary Figure 1. Correlation i) and Bland–Altman plot ii) comparing combined data from Trial-1 and Trial-2 for exercise-associated magnitude of change for plasma cortisol (A), I-FABP (B), sCD14 (C), and LBP (D) concentration; total leukocyte (E) and neutrophil counts (F); total bacterially-stimulated elastase release (G) and elastase release per cell (H); and plasma IL-1β (I), TNF-α (J), IL-10 (K), and IL-1ra (L) concentration. Dotted line represents limits of agreement (±2 SD; 95% confidence interval), and the solid line represents mean bias between trials.

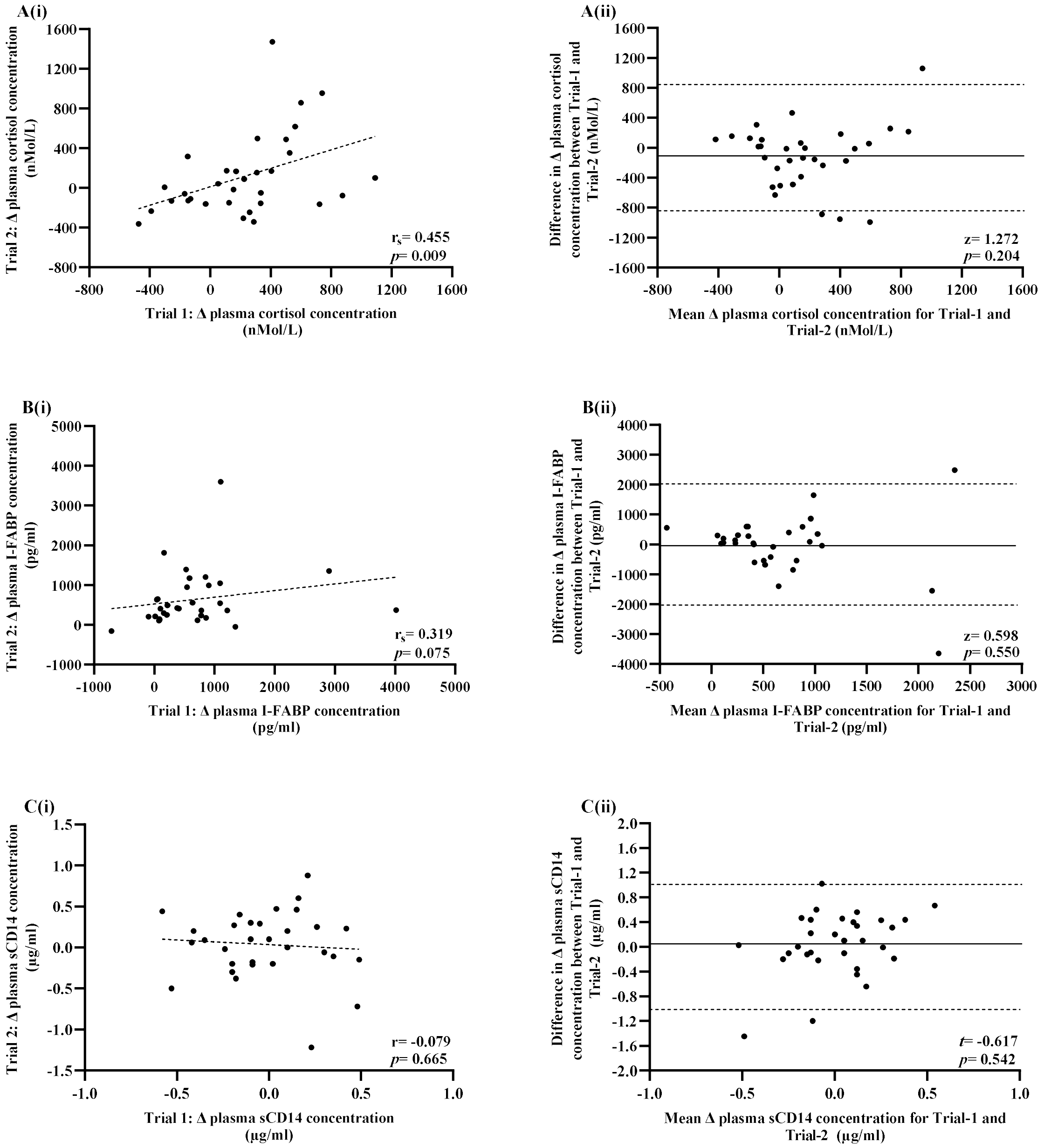

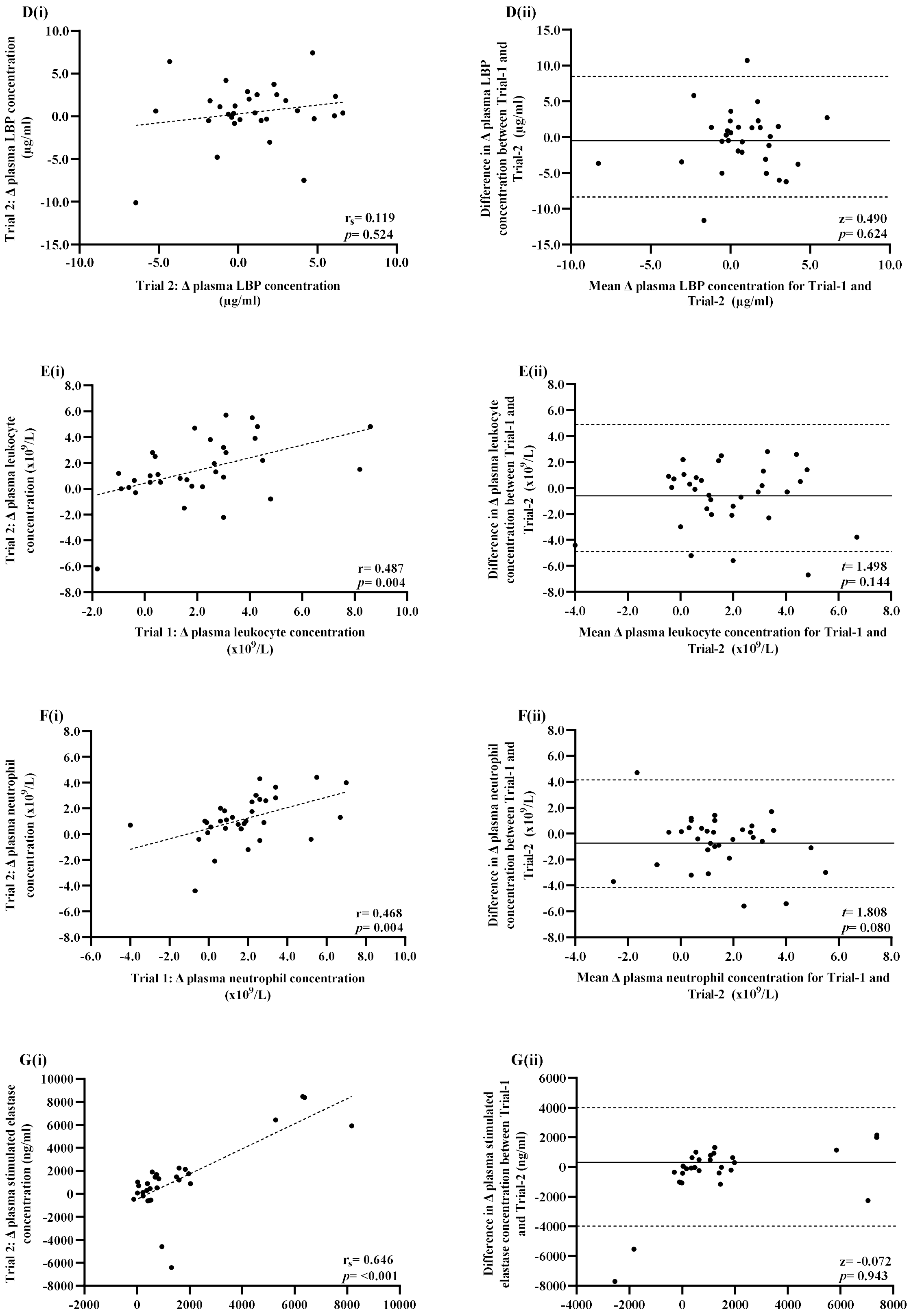
D(i)
Trial 2: Δ plasma LBP concentration (μg/ml)
Trial 2: Δ plasma LBP concentration (μg/ml)
r_s= 0.119
p= 0.524
D(ii)
Difference in Δ plasma LBP concentration between Trial-1 and Trial-2 (μg/ml)
Mean Δ plasma LBP concentration for Trial-1 and Trial-2 (μg/ml)
z= 0.490
p= 0.624
E(i)
Trial 2: Δ plasma leukocyte concentration ($x10^9$/L)
Trial 1: Δ plasma leukocyte concentration ($x10^9$/L)
r= 0.487
p= 0.004
E(ii)
Difference in Δ plasma leukocyte concentration between Trial-1 and Trial-2 ($x10^9$/L)
Mean Δ plasma leukocyte concentration for Trial-1 and Trial-2 ($x10^9$/L)
t= 1.498
p= 0.144
F(i)
Trial 2: Δ plasma neutrophil concentration ($x10^9$/L)
Trial 1: Δ plasma neutrophil concentration ($x10^9$/L)
r= 0.468
p= 0.004
F(ii)
Difference in Δ plasma neutrophil concentration between Trial-1 and Trial-2 ($x10^9$/L)
Mean Δ plasma neutrophil concentration for Trial-1 and Trial-2 ($x10^9$/L)
t= 1.808
p= 0.080
G(i)
Trial 2: Δ plasma stimulated elastase concentration (ng/ml)
Trial 1: Δ plasma stimulated elastase concentration (ng/ml)
r_s= 0.646
p= <0.001
G(ii)
Difference in Δ plasma stimulated elastase concentration between Trial-1 and Trial-2 (ng/ml)
Mean Δ plasma stimulated elastase concentration for Trial-1 and Trial-2 (ng/ml)
z= -0.072
p= 0.943

H(i)

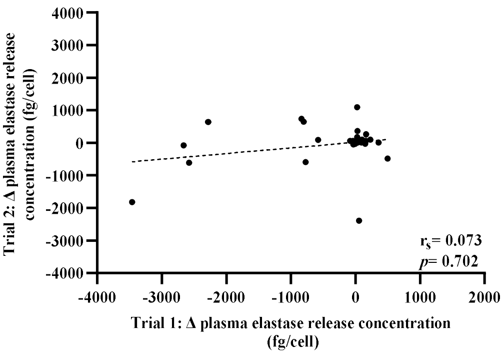

H(ii)

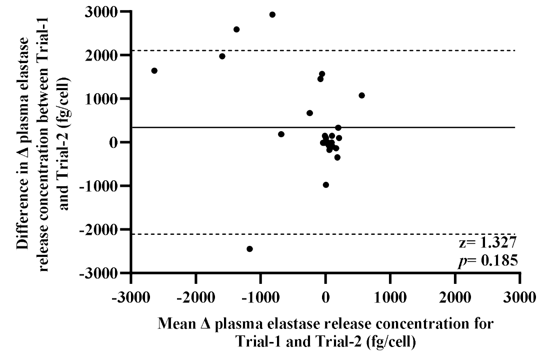

I(i)

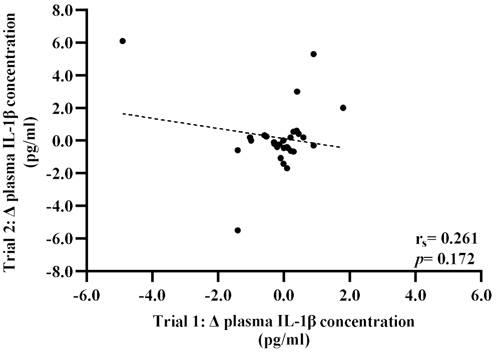

I(ii)

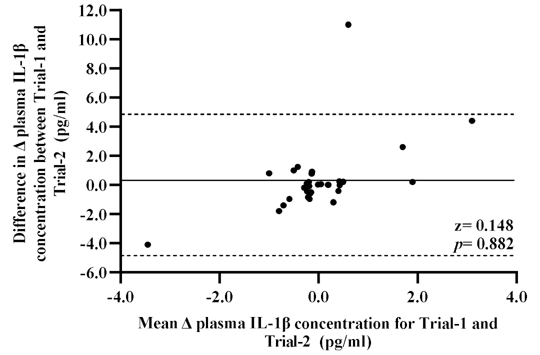

J(i)

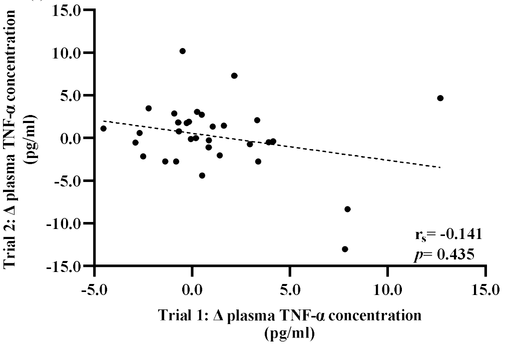

J(ii)

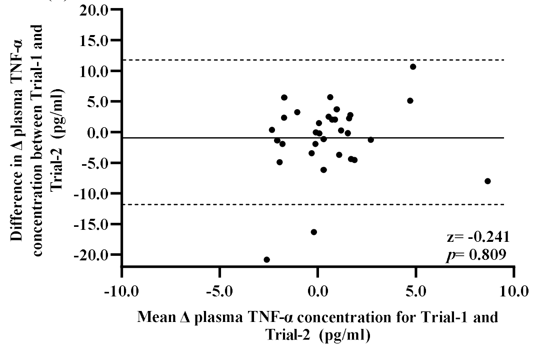

K(i)

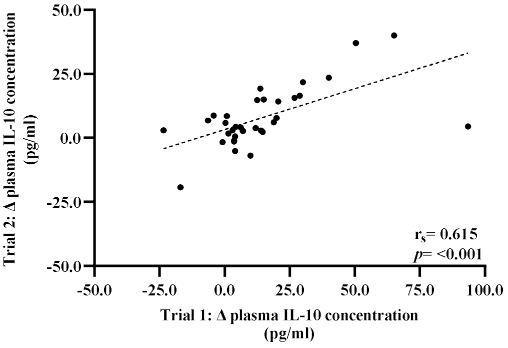

K(ii)

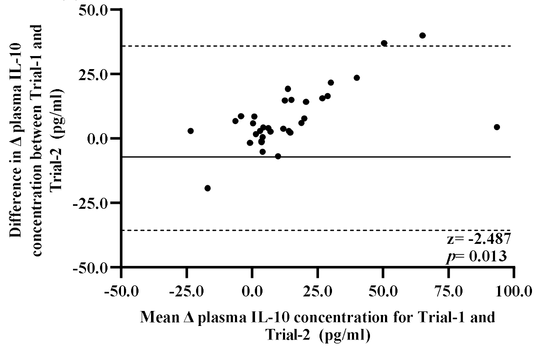

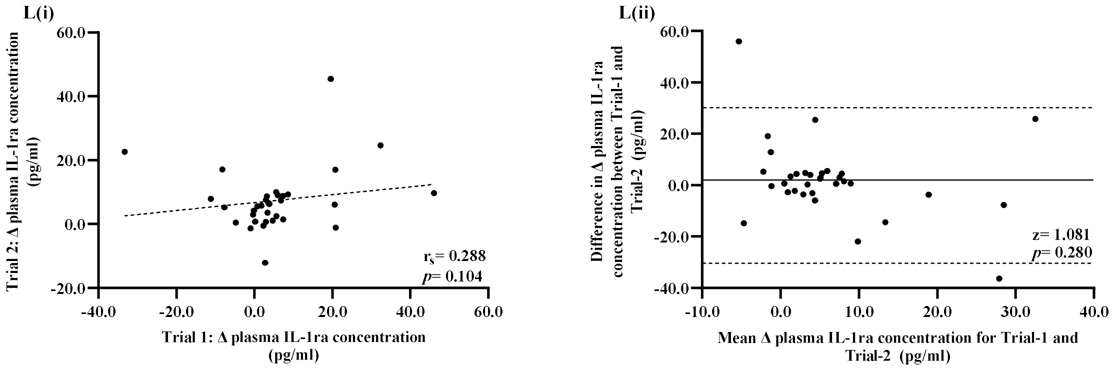
L(i)
Trial 2: Δ plasma IL-1ra concentration (pg/ml)
Trial 1: Δ plasma IL-1ra concentration (pg/ml)
rs= 0.288
p= 0.104
60.0
40.0
20.0
0.0
-20.0
-40.0
-20.0
0.0
20.0
40.0
60.0
L(ii)
Difference in Δ plasma IL-1ra concentration between Trial-1 and Trial-2 (pg/ml)
Mean Δ plasma IL-1ra concentration for Trial-1 and Trial-2 (pg/ml)
z= 1.081
p= 0.280
60.0
40.0
20.0
0.0
-20.0
-40.0
-10.0
0.0
10.0
20.0
30.0
40.0
